# *Tuber indicum* colonization enhances plant drought tolerance by modifying physiological, rhizosphere metabolic and bacterial community responses in *Pinus armandii*

**DOI:** 10.3389/fpls.2025.1642071

**Published:** 2025-10-28

**Authors:** Lanlan Huang, Shanping Wan, Yong Liu, Jian Zhan, Faming Zhang, Hui Yang, Fengming Zhang, Xuedan Xie, Xiaofei Shi, Yanliang Wang, Fuqiang Yu

**Affiliations:** ^1^ College of Yunnan Rural Revitalizing Education, Yunnan Open University, Kunming, Yunnan, China; ^2^ The Germplasm Bank of Wild Species &Yunnan Key Laboratory for Fungal Diversity and Green Development, Kunming Institute of Botany, Chinese Academy of Sciences, Kunming, Yunnan, China; ^3^ College of Resources and Environment, Yunnan Agricultural University, Kunming, Yunnan, China; ^4^ Interdisciplinary Research Center for Agriculture Green Development in Yangtze River Basin, College of Resources and Environment, Southwest University, Chongqing, China; ^5^ Key Laboratory of Chemistry in Ethnic Medicinal Resources, School of Ethnic Medicine, Yunnan Minzu University, Kunming, Yunnan, China; ^6^ Herbarium of Kunming Institute of Botany, Chinese Academy of Sciences, Kunming, Yunnan, China; ^7^ Guizhou Kangqunyuan Biotechnology Co., Ltd., Liupanshui, China

**Keywords:** plant drought resistance, mycorrhizosphere, bacterial communities, photosynthetic pigments, malondialdehyde

## Abstract

*Tuber indicum* has been extensively investigated in the fields of ecology, mycorrhizal synthesis with various plant species, and cultivation. Its impacts on plant physiological and metabolic responses under different water regimes remain unclear. Here, *T. indicum* colonized *Pinus armandii* seedlings were used to assess plant physiological responses, rhizosphere metabolomic profiles, and microbial community dynamics under differential water regimes. The results showed that *T. indicum* colonization significantly increased the contents of chlorophyll a and total pigments, but decreased the malondialdehyde content in the leaves under moderate drought stress. Under moderate and severe drought stresses, the diversity of the mycorrhizosphere bacterial community in the *T. indicum* - colonized group was significantly lower than that in the control group. Meanwhile, the bacterial community structures were similar under various drought conditions. Metabolic analysis revealed that carbohydrates and their derivatives were the most upregulated, while amino acids and their derivatives were the most downregulated differential expressed metabolites in *T. indicum* - colonized seedlings, compared to the control group. The relative abundance of *Nocardioides albus* has a significant positive correlation with trans-2-hexenal, and trans-2-hexenal shows a significant negative correlation with the malondialdehyde content and a significant positive correlation with the content of photosynthetic pigments, implying that *N. albus* might play an important role in regulating plant drought tolerance. Overall, these results suggest that *T. indicum* colonization could modulate the host plant’s physiological, mycorrhizosphere bacterial community and metabolic responses to enhance plant drought tolerance. These findings support the application of *T. indicum* mycorrhizal seedlings in drought-prone areas for ecological restoration and truffle cultivation.

## 1 Introduction

Drought stress is increasing worldwide, and it is a major environmental challenge that significantly impairs plant survival, growth, and productivity (Fahad et al., 2017; Cabello et al., 2023). Drought can directly limit tree growth and survival by restricting water use and photosynthesis, and can also indirectly impair root nutrient uptake through disrupting the mycorrhizal symbiosis between tree roots and soil fungi (Hammond et al., 2022; Hartmann et al., 2022; Xie et al., 2024). Soil fungi are crucial drivers for organic matter dynamics as well as nutrient release and uptake in coniferous forest ecosystems, while drought may alter the composition of soil fungal communities (Solly et al., 2017; Castaño et al., 2018). Furthermore, drought stress acts as a key modulator of plant phenotypic plasticity, driving adaptive changes in root architecture and carbon allocation strategies (Sardans and Penuelas, 2013).

Ectomycorrhizal fungi (ECMF) are the predominant symbionts in temperate forests, they can form ectomycorrhiza (ECM) with fine roots to improve plant water and nutrient acquisition, and in turn, acquire carbohydrates from trees (Smith and Read, 2008; Castaño et al., 2018). Moreover, they can also improve soil structure and porosity by forming and stabilizing soil aggregates and organic matter (Querejeta, 2017). Ectomycorrhizae (mantle, rhizomorphs, emanating hyphae and Hartig net) can expand the effective soil volume and root absorption area of plants, the formation of emanating hyphae enable plants to utilize water that relatively far from the root system (Tedersoo and Bahram, 2019). Besides, the formation of ectomycorrhizae can improve the hydraulic conductivity of plant roots-soil, increase the water conductivity rate, and increase the expression level of aquaporin genes, ensuring the normal circulation of water in plants under drought conditions (Warren et al., 2008; Navarro-Ródenas et al., 2013). These coordinated adaptations at both structural and molecular levels synergistically maintain plant water homeostasis under drought stress.

ECMF play a crucial role in enhancing plant drought tolerance through the modulation of root exudates and microbial interactions. They alter root exudate composition, promoting the secretion of organic acids and metabolites that improve soil nutrient availability and water uptake efficiency (Das and Sarkar, 2024; Wang et al., 2024). They also stimulate the release of plant hormones like abscisic acid and jasmonic acid, which regulate stomatal closure, reduce transpiration, and enhance water conservation (Abdelaziz et al., 2025). Moreover, ECMF optimize the rhizosphere microbial community, fostering beneficial interactions with nitrogen-fixing and phosphate-solubilizing bacteria. This leads to improved nutrient cycling and soil structure, including the formation of stable soil aggregates that increase porosity and water retention (Berrios et al., 2024; Attia et al., 2025).

Truffle (*Tuber* spp.) are ECMF with hypogeous fruiting bodies, which need to develop a symbiosis with host plants to complete their lifecycles (Kües and Martin, 2011). Due to its good fragrance and taste, some truffle species (as for instance *Tuber magnatum*, *T. melanosporum*, *T. borchii*, and *T. aestivum*) are of considerable economic and commercial importance (Monaco et al., 2022). Moreover, their ECM can also release carbon- (C) and nitrogen-(N) containing exudates including amino acids, organic acids and enzymes to mobilize less available nutrients form the soil (Wang and Lambers, 2020). *Tuber* ectomycorrhizae can alter their hosts’ root carbon exudation and metabolic profiles, as well as mycorrhizosphere microbial communities (Giorgio et al., 2022; Berrios et al., 2024). Additionally, soil bacterial communities play important roles in plant resistance to biotic and abiotic stresses (Bulgarelli et al., 2013). *Tuber indicum* is the first successfully cultivated truffle in China, and also the main commercial truffle species in China, particularly in the southwest (Wang et al., 2020). It shares similar morphological characteristics and has a close phylogenetic relationship with *T. melanosporum* (Murat et al., 2008). In recent years, its phylogeny, population genetics, mycorrhizal synthesis, biological characteristics, ecological values and edible and medicinal values have been widely studied (Lu et al., 2021; Wang et al., 2022; Huang et al., 2023; Elkhateeb et al., 2024). It can be associated with more than 20 tree species belonging to families such as Fagaceae, Juglandaceae, Pinaceae and Salicaceae. Previous studies have demonstrated that *T. indicum* colonization could significantly increase leaf photosynthetic rate, leaf P concentration and rhizosphere acid phosphatase activity, as well as richness of rhizosphere bacterial communities in *Castanopsis rockii* seedlings (Huang et al., 2020, 2023). However, whether it can improve the resistance of host plants to drought stress is still unknown.

*T. indicum* is not only an ecologically important ectomycorrhizal fungus but also holds substantial economic value as one of the most widely cultivated commercial truffle species in China, particularly in southwest regions (Wang, 2012; Wang et al., 2020). Its fruiting bodies are highly valued in local and international markets for their unique flavor and nutritional properties, with a well-established cultivation industry supporting rural livelihoods (Lu et al., 2021; Elkhateeb et al., 2024). This dual role-ecological facilitator and economic crop-makes it a promising candidate for applications in drought-prone areas, where ecological restoration and sustainable agriculture are often prioritized.

From an economic perspective, the use of *T. indicum* mycorrhizal seedlings offers a cost-effective strategy for ecological restoration and truffle cultivation. Unlike expensive chemical fertilizers or irrigation systems, mycorrhizal inoculation can be integrated into existing seedling production protocols with minimal additional input (Huang et al., 2022). Moreover, the potential for concurrent truffle production in restored areas creates an additional revenue stream, enhancing the economic viability of ecological projects. This dual benefit-improving plant drought tolerance for restoration while generating economic returns through truffle harvesting-positions *T. indicum* as a sustainable solution for drought-prone regions where agricultural and ecological goals intersect.

The objective of this study was to assess how *T. indicum* colonization affects *P. armandii* seedlings’ physiological traits, rhizosphere bacterial communities, and metabolites under differential water regimes. We hypothesized that (1) the colonization of *T. indicum* will improve the physiological performance of *P. armandii* under moderate and severe water stress; (2) *T. indicum* ectomycorrhization will improve the diversity and abundance of rhizosphere bacterial communities under water stress; (3) *T. indicum* ectomycorrhization will alter the root metabolism of *P. armandii*, such as the metabolism of amino acids and sugars to obtain a large number of osmotic regulatory compounds to resist the damage from drought stress. The overarching goal was to understand the host plant physiological responses caused by mycorrhization with *T. indicum* under different water regimes, which will provide useful information for *T. indicum* cultivation, and application of the mycorrhizal seedlings in the ecological restoration of arid areas.

## 2 Materials and methods

### 2.1 Seedling cultivation and experimental setup

Seeds of *Pinus armandii* were purchased from local market in Kunming, Yunnan, China. Before germination, seeds were first washed in tap water and surface- sterilized in 30% H_2_O_2_ for 30 min. After being thoroughly rinsed in distilled water, seeds were germinated in a large plastic crate, which was lined with a cotton mesh that held a sterilized growth substrate (perlite, vermiculite and water = 1:1:1, v: v), at a greenhouse in the Kunming Institute of Botany (KIB) under natural environment.

Ascomata of *Tuber indicum* were purchased from local market in Kunming, Yunnan, China. These were identified by morphological and molecular techniques, then sliced and dried at room temperature for over 72 h and stored in plastic boxes at 4°C until use. The synthesis of ectomycorrhizal seedling was performed as described by Huang et al. (2022). In brief, three months after seed germination, seedlings with similar size were selected, washed and transplanted into sterilized substrate (peat: vermiculite: perlite: water = 2:3:1, v:v; pH 7, adjusted with calcium carbonate and magnesium carbonate). The inocula of *T. indicum* were obtained by blending the ascomata, which was soaked in non-sterile distilled water for 24 h at 4°C. Each seedling was inoculated with 5 mL spore slurry (~1 ×10^6^ spores) in May 2022. Meanwhile, each un-inoculated control seedling received 5 mL of sterilized spore slurry. In a random location arrangement growing at the greenhouse at KIB, a total of 100 P*. armandii* seedlings were cultivated: 50 *T. indicum* and 50 control seedlings, all the seedlings were cultivated in 688-mL square plastic pots (13.2 × 6.4 × 9.1 cm) with one plant per pot. Seedlings were watered two or three times per week with tap water during cultivation. No fertilizer was applied.

Six months after inoculation, *T. indicum* mycorrhizae colonization rate were examined under a stereomicroscope (Leica S8AP0, Leica Microsystems, Wetzlar, Germany) according to the method of Murat 2015, 36 mycorrhizal seedlings with 50-70% mycorrhizal colonization rate and 36 uninoculated seedlings were selected for the drought treatment.

According to the classification of plant water gradient proposed by Hsiao (1973), four water gradients were set: (1) well: 75-80% (percentage of soil field water capacity); (2) mild: mild stress 60-65%; (3) moderate: moderate stress 40-50%; (4) severe: severe stress 25-35%. The complete crossing of these two factors created 8 different group, with 9 replicates for each group (4 water treatments × 2 ECM (with or without ECMF) × 9 replications = 72 pots). The experiment was initiated in December, 2022 and was lasted for 40 days. Water conditions was controlled by weighing method from 9:00-10:00 every morning, at the culture room with relatively constant light (14/10, day/night), temperature (22-25°C) and humidity (40%-60% RH) in KIB.

### 2.2 Determination of physiological parameters

The investigated physiological parameters of *P. armandii* seedlings included leaf photosynthetic pigment content, leaf malondialdehyde (MDA), root proline content, and root activity. Using ethanol extraction process to determinate leaf photosynthetic pigment content. About 0.1 g fresh mature leaves from each plant were carefully sampled using tweezers and scissors, then immersed in 96% ethanol solution and placed in dark for 24 h. Leaf photosynthetic pigment content (Chlorophyll a, chlorophyll b and carotenoids) was measured by a Bio Tek Cytation multifunctional enzyme marker (Bio Tek, America) at 665 nm, 649 nm and 470 nm, the injection volume was 300 μL. The leaf photosynthetic pigment content were determined according to:


$$\text{Pigment content }({\text{mg}\cdot \text{g}}^{-1}\text{FM})=\text{C}\times \text{VT}\times \text{n}/\text{FM}\times 1000$$

Where C is pigment concentration (mg·g^-1^), FM is fresh weight, VT is total volume of extract, n is dilution ratio.

The content of malondialdehyde (MDA) in leaf can reflect the degree of membrane lipid peroxidation. The malondialdehyde (MDA) content in leaves was quantified using the thiobarbituric acid reactive substances (TBARS) assay with trichloroacetic acid (TCA) extraction, following the protocol established by Hodges et al. (1999). About 0.3 g fresh leaves from each plant were sampled, then immersed in 2 mL 0.05 mol·L^-1^ phosphate buffer (refrigerated), and grind into homogenate using a high-flux tissue mill (Scientz-48, Schneider Electric, Beijing, China). After homogenate was transferred to the tube, 3 ml 0.05mol·L^-1^ phosphate buffer was used to flush the mortar for three times, and combined the extract. Then add 5 mL 0.5% thiobarbituric acid solution to the extract and blending. Put the homogenate into the boiling water bath for 10 min (from the beginning of the small bubble in the tube homogenate), take out the tube and put in the cold water bath immediately. After the homogenate was cooled, centrifuge at 3000 g for 15 min, take the supernatant and measure its volume. The light absorption values were measured using a BioTek microplate reader (Cytation, USA) at 532 nm, 600 nm and 450 nm, 0.5% thiobarbituric acid solution as blank. The calculation method of malondialdehyde is as follows:


$$\begin{array}{c} \text{MDA }\left({\text{mmol}\cdot \text{g}}^{-1}\text{FW}\right)\\ =\left[6.452\times \left(\text{D}532-\text{D}600\right)-0.559\times \text{D}450\right]\times \text{Vt}/\left(\text{Vs}\times \text{FW}\right) \end{array}$$

Where Vt is total volume of extract (mL), Vs is Volume of extracted liquid (mL) for determination, FW is Sample fresh weight (g).

The determination method of proline content in root system is as follows: After washing the fresh root system and cutting it up, take 0.2 g and put it into a centrifuge tube. Add 5 mL of 3% sulfosalicylic acid solution, and extract it in a boiling water bath for 10 minutes (while shaking). After cooling, centrifuge it at 3000 r/min for 10 minutes. The supernatant is the proline extract. Take 1 mL of the extract to a centrifuge tube, add 1 mL of water, 1 mL of glacial acetic acid, and 2 mL of acidic ninhydrin solution, and heat it in a boiling water bath for 60 minutes. The solution will turn red. After cooling, add 4 mL of toluene, vortex for 30 seconds, and let it stand for a while. Then, draw the red toluene proline solution from the upper layer into a cuvette, use toluene as a blank to adjust to 100, and measure the absorbance value by a Bio Tek Cytation multifunctional enzyme marker (Bio Tek, America) at 520 nm. Finally, obtain the concentration of proline in the determination solution from the standard curve, and then calculate the proline content according to the formula (Li, 2001).


$$\begin{array}{c} \text{Proline content}\left(\%\right)=\text{x}\times \left(\text{Vt}/\text{Vm}\right)/(\text{W}\times 10^{6})\times 100\%\\ =(\text{C}\times 5)/(\text{W}\times 10^{6})\times 100\% \end{array}$$

(x is the proline concentration (μg·mL^-1^) in the determination solution on the standard curve, that is, the C value; Vt is the total volume of the extract (mL); Vm is the volume of the extract used during the determination (mL); W is the fresh weight of the sample (g)).

The determination method of root activity is the 2,3,5-Triphenyltetrazolium chloride (TTC). Firstly, a standard curve is established using the TTC standard substance. Weigh 0.5 g of the root tip sample and place it in a 10 mL beaker. Add 10 mL of an equal-volume mixture of 0.4% TTC solution and phosphate buffer solution to fully immerse the roots in the solution. Conduct dark heat preservation at 37°C for 3 hours. After that, add 2 mL of 1 mol·L^-1^ sulfuric acid to stop the reaction. Then, take out the roots, dry the moisture, and grind them together with 4 mL of ethyl acetate and a small amount of quartz sand in a mortar to extract formazan. Transfer the red extract to a test tube, and wash the residue three times with a small amount of ethyl acetate and transfer them all to the test tube. Finally, add ethyl acetate to make the total volume 10 mL. Conduct colorimetry at a wavelength of 485 nm using a Bio Tek Cytation multifunctional enzyme marker (Bio Tek, America) with the blank test as the reference to measure the absorbance. Using the standard curve to find out the amount of tetrazolium reduction. Finally, according to the formula:


$$\begin{array}{c} \text{Tetrazolium reduction intensity }\left({\text{mg}\cdot \text{g}}^{-1}\left(\text{fresh root weight}\right)\cdot {\text{h}}^{-1}\right)\\ =\text{Amount of tetrazolium reduction }\left(\text{mg}\right)/\text{Root weight}\left(\text{g}\right)\times \text{Time}\left(\text{h}\right) \end{array}$$

### 2.3 Mycorrhizosphere bacterial DNA extraction and 16S rRNA sequencing

DNA of the rhizosphere substrate of 0.2 g was extracted using the soil DNA extraction kit PowersoilTM (MoBio, USA). The V3-V4 fragments of the bacterial 16S rRNA gene were amplified using the forward primer 338F (5′-ACTCCTACGGGAGGCAGCA-3′) and the reverse primer 806R (5′-GGACTACHVGGGTWTCTAAT-3′). The purified amplicons were pooled, and sequenced on the Illumina MiSeq platform Miseq-PE250 (Personalbio, China). Sequences were processed using the QIIME 2 (Quantitative Insights into Microbial Ecology) pipeline following the steps of raw read quality control, paired-end clean read assembly, and raw tag quality control. Firstly, the exclusions of fuzzy base N and sequence lengths< 160 bp. Second, removal for the sequences with a mismatched base number > 1 of the 50 end primer, and the sequences with >8 identical consecutive bases. Finally, deletion of chimeric sequences by filtering sequences on the VSEARCH software. To improve sequencing accuracy and avoid overestimation of bacterial diversity, singletons (sequences that occurred only once in dataset) were removed from downstream analyses. Sequences with similarity ≥ 97% obtained were combined into an operational taxonomic unit (OTU) (Edgar, 2010). OTUs taxonomic cluster was processed via searching reads against the Greengenes (for bacterial OTUs) database. OTUs with abundance<0.001% were removed from final analysis. The remaining OTUs were grouped according to their assigned taxonomic levels. On the Personalbio^®^ Genescloud platform, based on Bray-Curtis distance, dissimilarity distance, observed species, Simpson, Shannon, and Chao1 diversity indices, as well as principal coordinate analysis (PCoA) and Venn diagrams of unique OTUs were obtained to analyze the differences in bacterial communities among the sample groups. The raw sequence data has been deposited in the NCBI Sequence Read Archive database under the bioproject identifier PRJNA1309842. In this assay, the mycorrhizosphere soil samples from three plants were pooled as one sample.

### 2.4 Mycorrhizosphere metabolites

Extraction of mycorrhizosphere metabolites: After taking the plants out of the substrate, gently wash the substrate on the root surface with sterile deionized water, and then wash it with deionized water three times repeatedly. Culture the cleaned plants with 50 mL of deionized water for 24 hours, and wrap the culture bottle with tinfoil to ensure that the roots are protected from light. Mix the enrichment solutions of the three individual plants, filter, and take 1 mL of the sample and freeze-dry it in a freeze dryer; add 100 mL of 80% methanol to dissolve the residue, vortex and shake, stand still in an ice bath for 5 minutes, centrifuge at 15000 xg and 4°C for 15 minutes, take a certain amount of the supernatant and dilute it with mass spectrometry grade water until the methanol content is 53%; centrifuge under the same conditions for 15 minutes, and collect the supernatant for on-machine analysis.

The ultra-high performance liquid chromatography-mass spectrometry (liquid chromate-graph-mass spectrometer, LC-MS) technology is adopted, and the targeted-like metabolomics research is carried out based on the high-sensitivity SCIEX QTRAP ^®^ 6500+ mass spectrometry platform. The mobile phases used are as follows: the aqueous phase is ultrapure water (with 0.1% formic acid added), and the organic phase is acetonitrile (with 0.4% formic acid added). The elution gradient is as follows: at 0 min, it is water:acetonitrile (98:2, V/V); at 2.0 min, it is eluted with water:acetonitrile (98:2, V/V); at 15.0 min, it is water:acetonitrile (0:100, V/V); at 17.0 min, it is water:acetonitrile (0:100, V/V); at 17.1 min, it is water:acetonitrile (98:2, V/V); and at 20.0 min, it is water:acetonitrile (98:2, V/V). The chemical reagents used for sample extraction and analysis all belong to preparations of analytical grade or chromatographic grade.

A 10 μL sample was injected onto an Xselect HSS T3 chromatographic column (2.1 mm × 150 mm, 2.5 μm). The column temperature was maintained at 50°C, and the flow rate was 0.4 mL·min-1. The samples after chromatographic separation were subjected to the next step of mass spectrometry analysis. The main parameters of the mass spectrometer include: the electrospray ionization (ESI) temperature is 550°C, the mass spectrometry voltage is 5500 V, the curtain gas (CUR) is 35 psi, and the collision-activated dissociation (CD) parameters are set to medium. The above work was completed by Beijing Novogene Technology Co., Ltd. The identification of metabolites is based on the qualitative analysis carried out in the novoDB (novogene database) self-built by Novogene, and the quantitative analysis is carried out using the SCIEX OS V1.4 software.

### 2.5 Statistical analysis

Statistical analysis of the obtained data was carried out using the data statistical analysis software R (4.2.2) and SPSS Statistics 26, results are presented as mean ± standard deviation (SD). The SIMCA software was used to conduct OPLS-DA multivariate statistical analysis on the normalized data substrate. The VIP (variable importance in the projection) value of multivariate statistical analysis and the T-test P value of univariate statistical analysis were used to determine the significantly different metabolites between different comparison groups (VIP value>1, FC>1.2 or FC<0.833, and P value<0.05). The differentially expressed metabolites were qualitatively hierarchical clustered and submitted to KEGG (www.genome.jp/kegg/) and MetaboAnalyst (www.metaboanalyst.ca/) for metabolic pathway analysis. Spearman correlation analysis was used to analyze the bacterial OTUs in the top 20 of relative abundance and the metabolites in the top 20 peaks. The statistical significance of Spearman correlation was determined by pairwise comparisons. Two-way ANOVAs were used to determine main effects of ECM, water regimes, and their interaction on all parameters. Student’s t-test was used to determine the significant differences between treatments.

## 3 Results

### 3.1 Effect of mycorrhization on plant physiological responses under different water regimes

**Figure 1:**
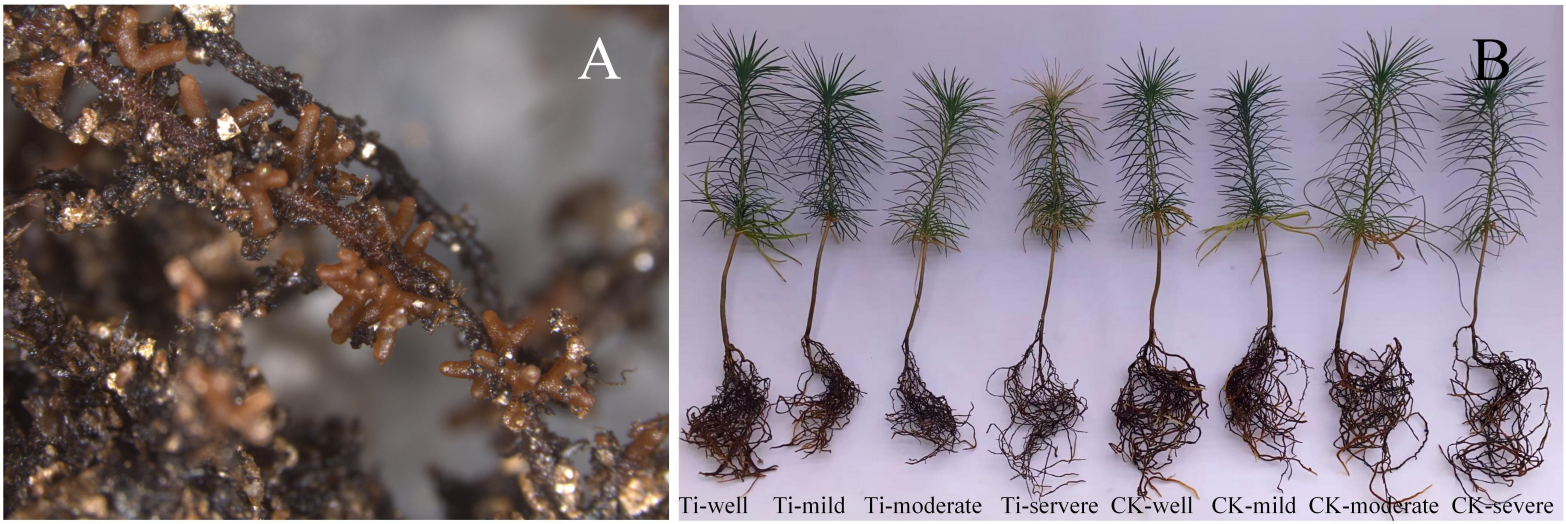
The effect of ectomycorrhiza on host plant growth under different water regimes. **(A)** Ectomycorrhizae of *Tuber indicum* associated with *Pinus armandii*. **(B)** The plant growth of *T. indicum* (Ti) colonized and un-inoculated (CK) seedlings under different water regimes. Treatments: well, 75-80% water holding capacity (WHC); mild, mild drought stress, 60-65% WHC; moderate, moderate drought stress, 40-50% WHC; severe, severe drought stress, 25-35% WHC.

**Figure 2:**
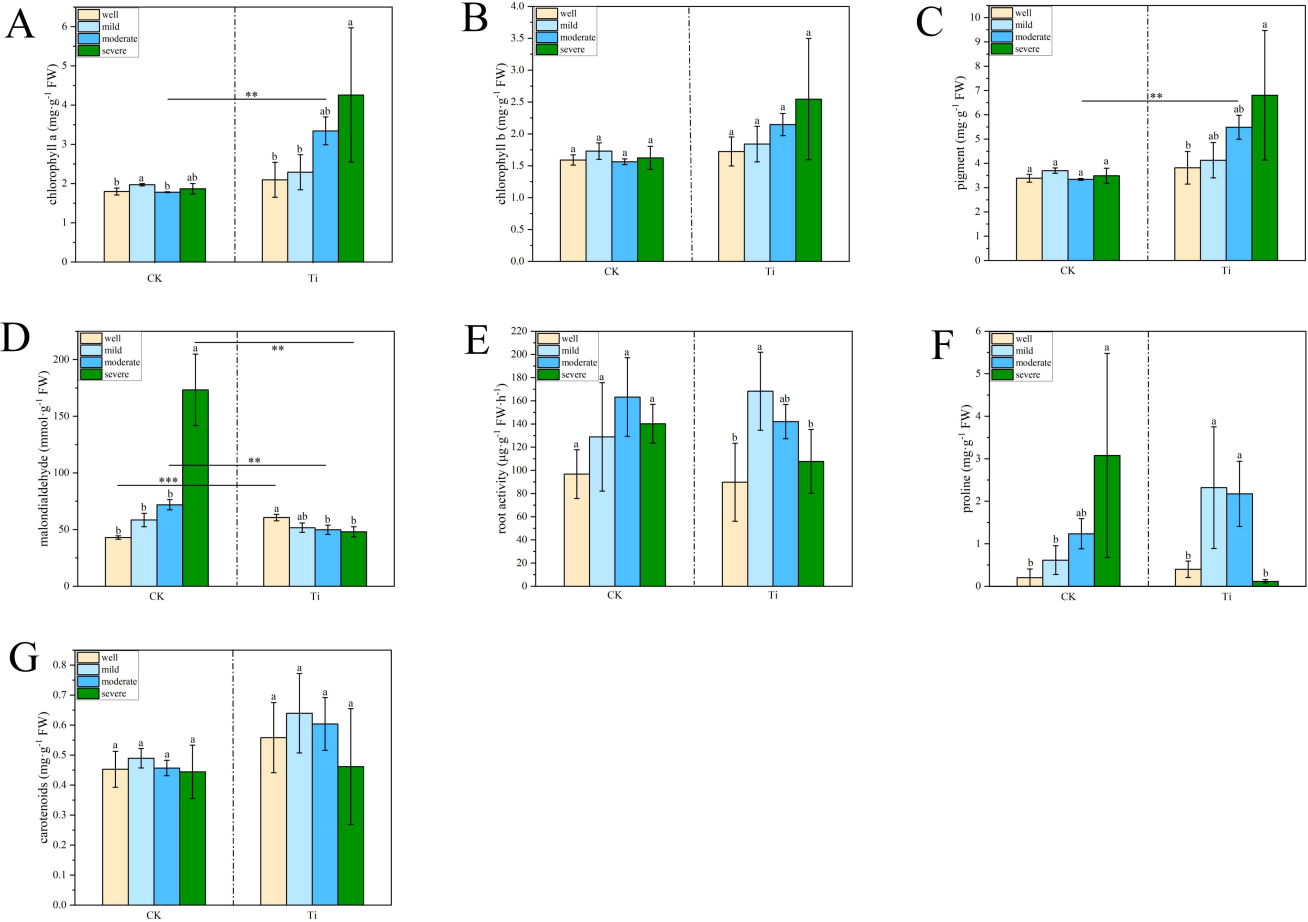
Ectomycorrhizal colonization altered plant physiological responses of *Pinus armandii* under different water regimes. **(A, B)** Impact of *Tuber indicum* (Ti) colonization on leaf chlorophyll a and b concentrations, and the effects of *Tuber indicum* (Ti) colonization on concentrations of leaf pigment **(C)**, leaf malondialdehyde **(D)**, root activity **(E)**, root proline **(F)**, and leaf carotenoid **(G)** of *Pinus armandii* seedlings under different water regimes. Bars show means ± SD. Different letters indicated significant differences across different water regimes within each group (mycorrhizal seedlings and control seedlings, respectively); *P < 0.05; **P < 0.01 and ***P < 0.001 indicates significant differences between mycorrhizal seedlings and the control samples under the same water regime.

After 40 days of water control, the growth of mycorrhizal and seedlings under different water regimes is shown in **Figures 1A, B**. The concentration of chlorophyll a and total photosynthetic pigments in the *T. indicum* colonization group were significantly higher when the water holding capacity (WHC) was 25-35% than those of 75-80%. At the same time, the malondialdehyde concentration in the leaves is also significantly lower than that in the group under WHC of 75-80% (**Figures 2A, C, D**). In addition, when the WHC of the substrate is 40-50%, the concentration of chlorophyll a and total photosynthetic pigments in the *T. indicum* colonization group are significantly higher than those in the control group. At the same time, the malondialdehyde concentration in the leaves is significantly lower in the WHC range of 40-50% and 25-35% compared to the control group with the same WHC. Furthermore, when the WHC is 25-35%, the root activity and proline concentration of the roots colonized by *T. indicum* are significantly lower than those when the WHC is 60 - 65%, and the proline content is also significantly lower than that when the WHC is 40-50% (**Figures 2E, F**).

As shown in **Table 1**, most parameters except proline concentration were significantly affected by the ECMF colonization. Water regimes had significant effects on Chlorophyll a concentration and MDA concentration. Additionally, the interaction of water regimes and ECMF significantly affected Chlorophyll a concentration, MDA concentration and proline concentration.

**Table 1:** Significance of a two-way ANOVA analysis for measured parameters.

Physiological parameters	Water regimes	ECM	Water regimes×ECM
Chlorophyll a	*	**	*
Chlorophyll b	ns	*	ns
Carotenoids	ns	*	ns
Pigment	ns	**	ns
Malondialdehtde	***	***	***
Root activity	ns	*	ns
Proline	ns	ns	**

Significant effects are indicated for Water regimes, ectomycorrhzas and their interactions (ns, not significant; *P < 0.05; **P < 0.01; ***P < 0.001).

### 3.2 Mycorrhizosphere bacterial communities under different water regimes

**Figure 3:**
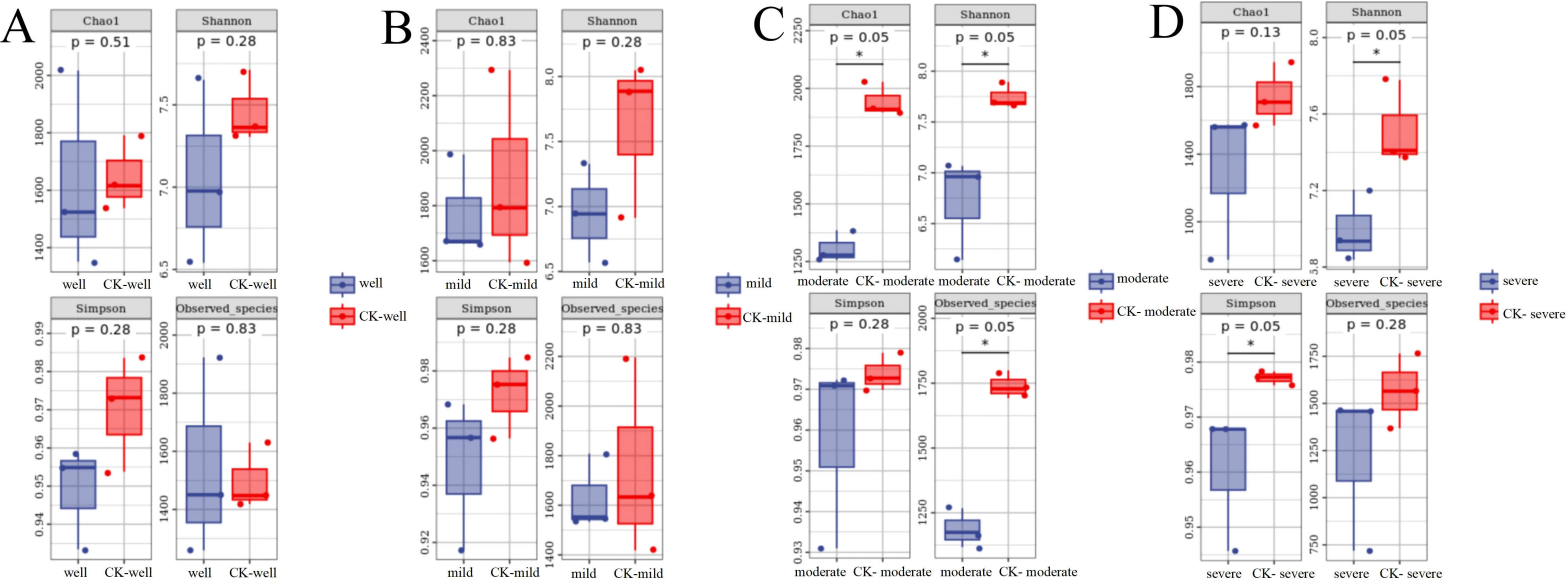
Moderate and severe drought stresses decreased the α diversity of bacterial communities in the ectomycorrhizosphere of *Pinus armandii* seedlings. Bacterial α diversity indicated by Observed species, Chao1 diversity, Shannon index and Simpson index between mycorrhizal samples and the control (CK) samples, under different water regimes: well, 75-80% water holding capacity (WHC) **(A)**; mild, mild drought stress, 60-65% WHC **(B)**; moderate, moderate drought stress, 40-50% WHC **(C)**; severe, severe drought stress, 25-35% WHC **(D)**. *P<0.05.

A total of 14,165 bacterial OTUs were obtained, belonging to 28 phyla, 80 classes, 309 families, 630 genera, and 1370 species. At the phylum level, the top three phyla with the highest relative abundances are Proteobacteria, Actinobacteria, and Bacteroidetes, and their average abundances in each group were 69%, 23%, and 5%, respectively (**Supplementary Figure 1**). The relative abundances of Bacteroides, Patescibacteria, Firicutes, and Armatimonadetes in the *T. indicum* colonization group were significantly higher than those in the control group. According to Chao1, Shannon, Simpson, and Observed species data (**Figure 3**), there was no significant differences in the α diversity of mycorrhizosphere bacterial communities,. However, in the analysis of the diversity of the mycorrhizosphere bacterial community of *T. indicum* colonized seedlings under the same WHC, it was found that when the WHC was 40 - 50%, the diversity of the bacterial community in the *T. indicum* colonization group was significantly lower than that in the control group under the indicators of Chao1, Shanon, and Observed species.

**Figure 4:**
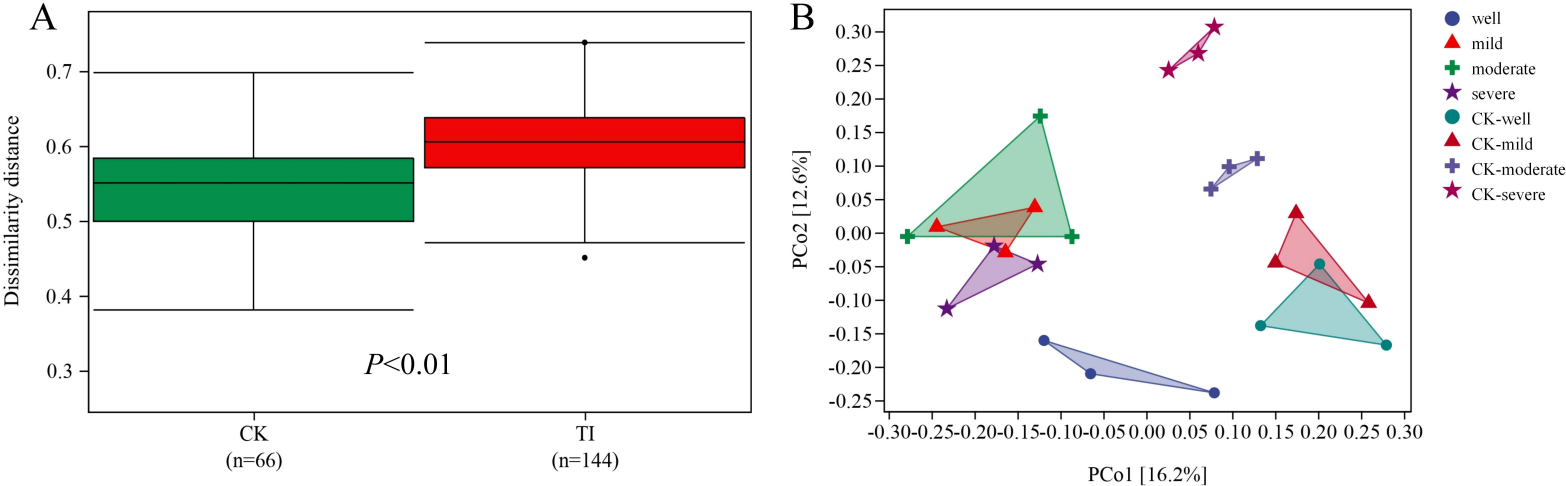
*Tuber indicum* colonization significantly changed the diversity of rhizosphere bacterial communities under drought stress conditions. **(A)** Dissimilarity distance, and **(B)** principal coordinate analysis (PcoA) of the bacterial communities in the rhizosphere of mycorrhizal (TI) and non-mycorrhizal (CK) seedlings under different water regimes: well, 75-80% water holding capacity (WHC); mild, mild drought stress, 60-65% WHC; moderate, moderate drought stress, 40-50% WHC; severe, severe drought stress, 25-35% WHC.

When the WHC is 25-35%, the Shanon and Simpson indices show that the diversity of the mycorrhizosphere bacterial community colonized by *T. indicum* is significantly lower than that of the control group (**Figures 3C, D**). For the β diversity analysis of each combination (**Figure 4**), it is found that there are significant differences in the mycorrhizosphere bacterial communities between the *T. indicum* colonization group and the control group (**Figure 4A**). Different water holding capacities also have obvious impacts on the mycorrhizosphere bacterial community. Especially for the control group, there are significant differences in the mycorrhizosphere bacterial community under different water holding capacities, while there is no obvious difference in the *T. indicum* colonization group among the water holding capacities of 60-65%, 40-50%, and 25-35% (**Figure 4B**). The hierarchical clustering analysis of each group of samples shows that the composition of the bacterial community is significantly different between the *T. indicum* colonization group and the control group. The community composition under the same WHC in the control group is more similar, while the differences in the community composition under different water holding capacities in the *T. indicum* colonization group are not obvious (**Supplementary Figure 2**).

Based on the petal diagram (**Supplementary Figure 3**), there were only 281 OTUs shared among all combinations, 2.0% of the total OTUs. Among them, when the WHC in the *T. indicum* colonization group was 40-50%, the unique bacterial OTUs were 7.9% of the total OTUs. However, when the WHC in the control group was 60-65%, the unique bacterial OTUs were 15.8% of the total OTUs. To further compare the differences in species composition among each group, a heat map was used to analyze the composition of the top 20 species in terms of species abundance in each group (**Supplementary Figure 4**). It was found that the abundances of the top 20 bacteria were relatively similar when the WHC in the *T. indicum* colonization group was 75-80% and the WHC in the control group was 75-80%, 60-65%, and 40-50%.

### 3.3 Mycorrhizosphere metabolites under different water regimes

A total of 856 metabolites were identified in this experiment, and these metabolites were classified into 21 types. They were mainly classified as amino acids and derivatives (17.3%), flavonoids (12.3%), organic acids and their derivatives (11.9%), carbohydrates and their derivatives (10.6%), lipids (7.8%), nucleotides and their derivatives (6.0%), and terpenoids (5.4%) (**Supplementary Figure 5**).

These 856 metabolites were mainly involved in four major metabolic pathways: cellular processes, environmental information processing, genetic information processing, and metabolism. Among them, the metabolites participating in metabolism were the most diverse, including global and overview (220), amino acid metabolism (94), carbohydrate metabolism (89), biosynthesis of other secondary metabolites (61), nucleotide metabolism (48), metabolism of cofactors and vitamins (29), lipid metabolism (28), energy metabolism (17), and metabolism of terpenoids and polyketides (14) (**Supplementary Figure 6**).

**Figure 5:**
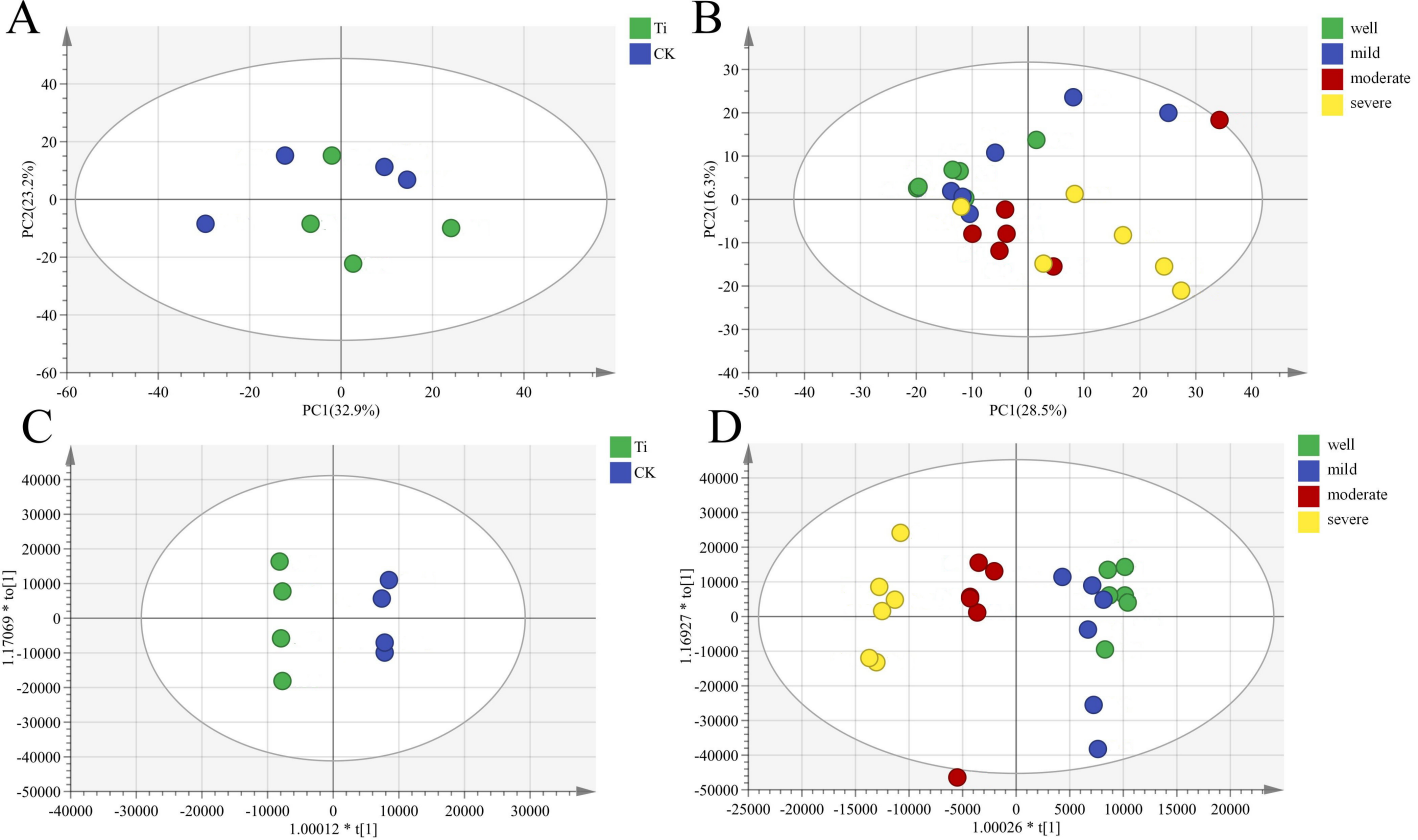
*Tuber indicum* colonization changed the root metabolic profiles of *Pinus armandii* seedlings under different water regimes. **(A, C)** Impact of *Tuber indicum* (Ti) colonization on root metabolites of mycorrhizal and non-mycorrhizal (CK) *Pinus armandii* seedlings; **(B, D)** Impact of *Tuber indicum* (Ti) colonization on root metabolites of mycorrhizal and non-mycorrhizal (CK) *Pinus armandii* seedlings under different water regimes (well, 75-80% water holding capacity (WHC); mild, mild drought stress, 60-65% WHC; moderate, moderate drought stress, 40-50% WHC; severe, severe drought stress, 25-35% WHC). **(A, B)** Principal component analysis (PCA); **(C, D)** Partial least squares-discriminant analysis (PLS-DA).

The principal component analysis (PCA) was used to observe the overall distribution trend among samples. The PCA analysis showed that there are differences in metabolites between the samples of the *T. indicum* colonization group and the control group, and there are also differences among different water gradients (**Figures 5A, B**). Partial least squares discriminant analysis (PLS-DA) can maximize the distinction between groups and is conducive to identifying differential metabolites. The results found that there are significant differences in metabolites between the samples of the *T. indicum* colonization group and the control group, and there are significant differences between the water holding capacities of 60-65%, 75-80% and 25-35% and 40-50% (**Figures 5C, D**). Evaluating the reliability of the PLS-DA model prediction mainly depends on R2 and Q2. If R2 and Q2 are closer to 1, it indicates that the model is more stable and reliable. In this experiment, the R2 and Q2 of metabolites in the *T. indicum* colonization group and non-mycorrhizal seedlings are both close to 1 (**Supplementary Figure 7**), indicating that the model is relatively stable and reliable.

**Figure 6:**
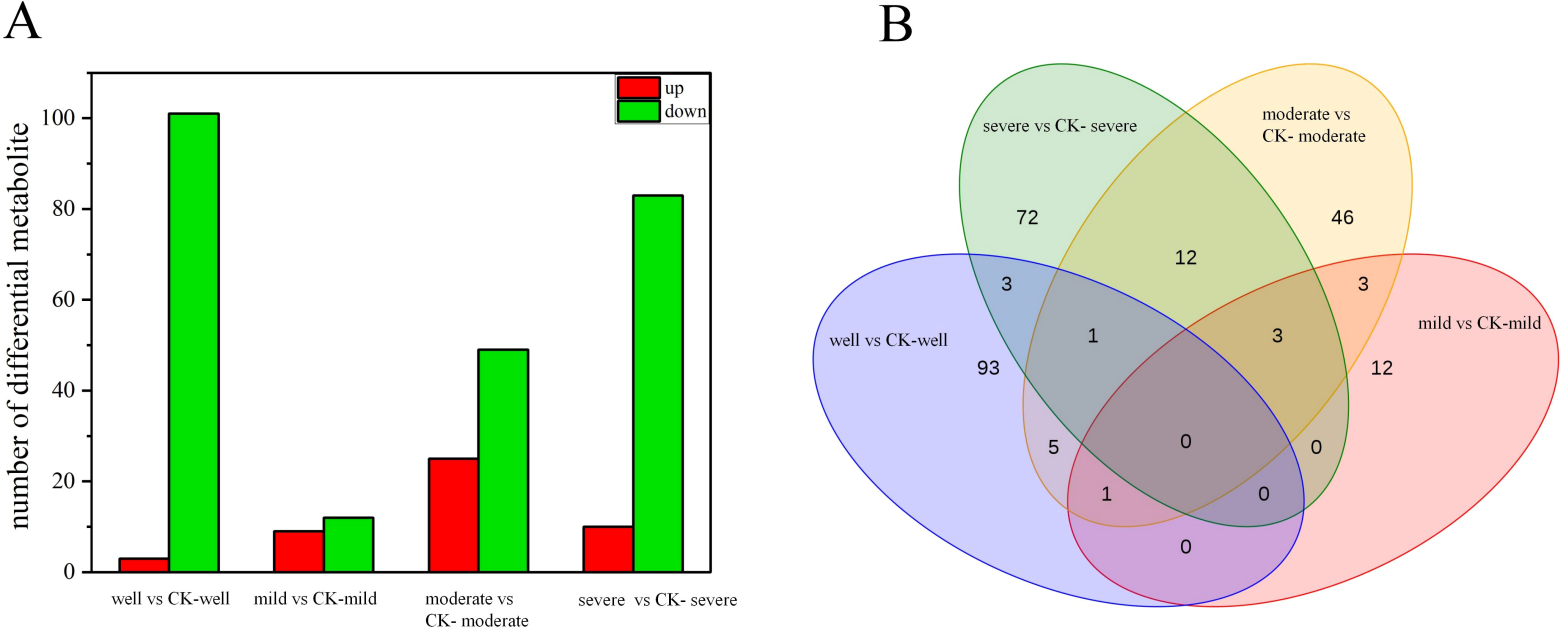
Differentially expressed root metabolites induced by *Tuber indicum* colonization. Number of differentially expressed metabolites **(A)** and Venn analysis **(B)** of shared root metabolites of *Pinus armandii* seedlings colonized or not colonized (CK) by *T. indicum*, under different water regimes: well, 75-80% water holding capacity (WHC); mild, mild drought stress, 60-65% WHC; moderate, moderate drought stress, 40-50% WHC; severe, severe drought stress, 25-35% WHC.

The expression of overall metabolites in the *T. indicum* colonization group and the control group under each WHC is shown in the volcano plot (**Supplementary Figure 8**). A total of 251 DEMs were identified in this experiment. The number of DEMs in the *T. indicum* colonization group and the control group is different under different water gradients. There are 103 DEMs when the WHC is 75-80%, 19 DEMs when the WHC is 60-65%, 71 DEMs when the WHC is 40-50%, and 91 DEMs when the WHC is 25-35% (**Figure 6A**). Among them, the most up-regulated expression is carbohydrates and their derivatives (13 DEMs), followed by organic acids and their derivatives (5 DEMs) and terpenoids (5 DEMs). The most down-regulated expression is amino acids and their derivatives (59 DEMs), followed by flavonoids and organic acids and their derivatives (33 DEMs each). Based on the Venn diagram of DEMs in four water gradients (**Figure 6B**), there are no common DEMs in the four groups. There are 12 common DEMs when the WHC is 25-35% and 40-50% (1 up-regulated and 11 down-regulated). The up-regulated one is lipids, and the down-regulated ones are organic acids and their derivatives (4 DEMs), organic heterocyclic compounds (2 DEMs), flavonoids, terpenoids, plant hormones, alkaloids and their derivatives, and amino acids and their derivatives (1 DEM each). Hierarchical cluster analysis of differentially expressed metabolites (DEMs) found that the *T. indicum* colonization group showed significant differences in metabolic levels different from the control group samples under different water holding capacities (**Supplementary Figure 9**).

**Figure 7:**
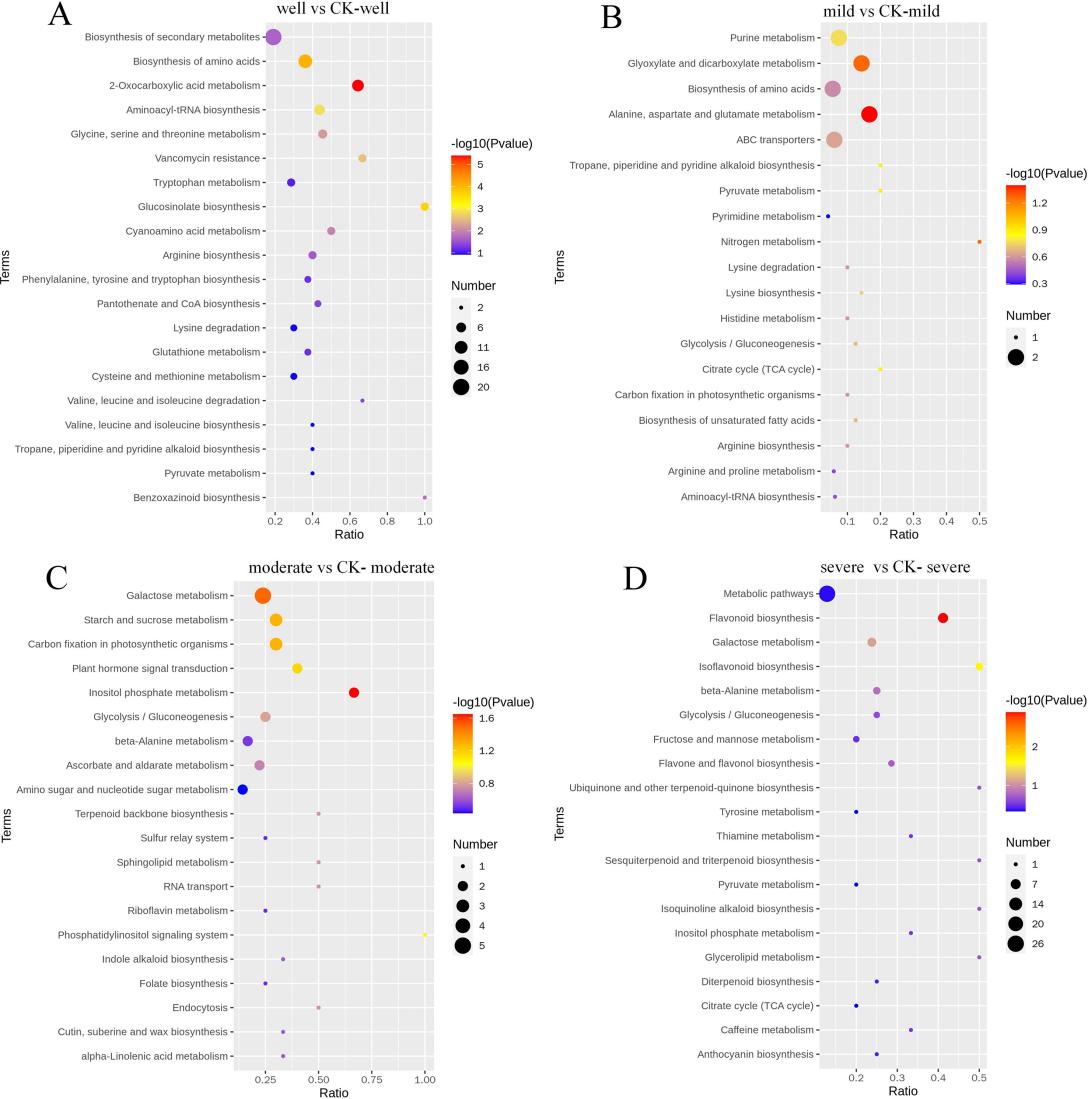
KEGG pathways of differentially expressed root metabolites between mycorrhizal and non-mycorrhizal plants under different water regimes. well, 75-80% water holding capacity (WHC) **(A)**; mild, mild drought stress, 60-65% WHC **(B)**; moderate, moderate drought stress, 40-50% WHC **(C)**; severe, severe drought stress, 25-35% WHC **(D)**. CK, the control samples.

By comparing the KEGG metabolic pathway annotation results of the identified DEMs, a KEGG bubble plot is made (**Figure 7**). When the WHC is 75-80%, a total of 40 DEMs are assigned to 39 pathways. Among them, DEMs are significantly enriched in 12 KEGG pathways. The largest category is the biosynthesis of secondary metabolites (Biosynthesis of secondary metabolites, 20 DEMs), followed by the biosynthesis of amino acids (Biosynthesis of amino acids, 13 DEMs) and 2-oxocarboxylic acid metabolism (2-Oxocarboxylic acid metabolism, 9 DEMs). When the WHC is 60-65%, a total of 9 DEMs are assigned to 19 pathways. Among them, 2 DEMs are significantly enriched in the alanine, aspartate and glutamate metabolism pathway. When the WHC is 40-50%, a total of 29 DEMs are assigned to 39 pathways. Among them, 2 DEMs are significantly enriched in the inositol phosphate metabolism pathway, and 5 are significantly enriched in the galactose metabolism pathway. When the WHC is 25-35%, there are 37 DEMs assigned to 31 metabolic pathways. Among them, 7 DEMs are significantly enriched in the flavonoid biosynthesis pathway, and 3 DEMs are significantly enriched in the isoflavonoid biosynthesis pathway.

### 3.4 Correlation analysis between measured parameters

**Figure 8:**
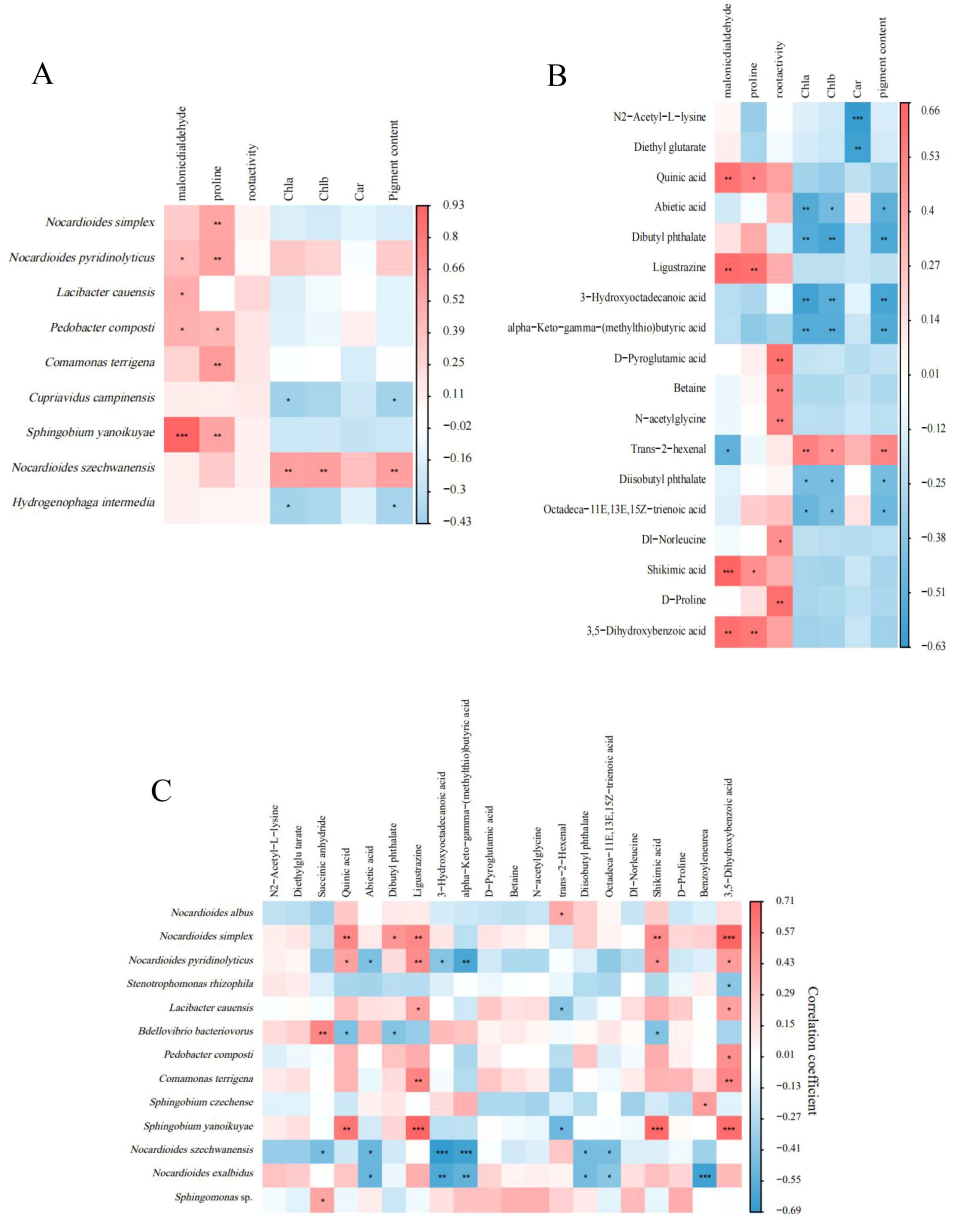
Heatmaps of Spearman correlation analyses between the 20 most prominent bacterial taxa and plant physiology parameters **(A)**; between the 20 most prominent metabolites and plant physiology parameters **(B)**; and between the 20 most prominent bacterial taxa and the 20 most prominent metabolites **(C)**. *P < 0.05; **P < 0.01; ***P < 0.001.

Correlation analysis was conducted on the measured physiological indicators and the top 20 species of mycorrhizosphere bacterial abundance as well as the top 20 metabolites in metabolite content (**Figure 8**). It was found that four species of bacteria (*Nocardiodes pyri dinolyticus*, *Lacibacter cauensis*, *Pedobacter composti*, *Sphingobium yanoikuyae*) had a positive correlation with malondialdehyde content, fivespecies of bacteria (*N. simplex*, *N. pyridinolyticus*, *P. composti*, *Comamonas terrigena*, *S. yanoikuyae*) had a significant positive correlation with proline, while *Nocardioides szechwanensis* had a significant positive correlation with photosynthetic pigment content, and *N. simplex* had a positive correlation with proline content (**Figure 8A**). In addition, the content of proline was significantly positively correlated with the content of four metabolites in the mycorrhizosphere. Root activity was significantly positively correlated with D-Pyroglutamic acid, Betaine, N-acetylglycine, Dl-Norleucine and D-Proline. Quinicacid, ligustrazine, shikimic acid, and 3, 5-Dihydroxybenzoic acid were all significantly positively correlated with leaf malondialdehyde and proline content. Trans-2-hexenal had a significant negative correlation with malondialdehyde and a significant positive correlation with photosynthetic pigments (**Figure 8B**). Spearman correlation analysis was conducted on the top 20 species of bacterial abundance in mycorrhizosphere samples of each group of plants and the top 20 metabolites in mycorrhizosphere metabolite content to obtain a correlation heat map (**Figure 8C**). The results showed that *Nocardioides albus* had a significant positive correlation with 2-hexenal. *Sphingomonas* sp. had a significant positive correlation with succinic anhydride. *Pedobacter composti* had a significant positive correlation with 3, 5-Dihydroxybenzoic acid. At the same time, *Comamona terrigena* had a significant positive correlation with two metabolites. *Nocardioides simplex* had a significant positive correlation with five metabolites. The analysis found that *N. albus* may reduce the formation of host malondialdehyde by increasing the secretion of hexenal in the root system and at the same time improve the photosynthetic capacity of plants; while *P. comosti* may change the drought resistance of plants by promoting the secretion of 3, 5-Dihydroxybenzoic acid in plants.

## 4 Discussion

Drought stress constitutes a critical environmental constraint on plant productivity, exacerbating global food security challenges through compromised agricultural outputs (Babst et al., 2019; Vilonen et al., 2022). Extensive research has elucidated the pivotal role of ECMF in mitigating drought and salinity stress via enhanced nutrient acquisition, photosynthetic optimization, and improved plant water-use efficiency (Liu et al., 2021). Notably, truffle species exhibit species-specific hydrological optima: *Tuber melanosporum* achieves maximal yields under moderate soil moisture (Büntgen et al., 2015), while *T. aestivum* demonstrates increased soil DNA detectability under progressive water stress (Todesco et al., 2019). Conversely, the white truffle *T. magnatum* thrives in hydrologically stable alluvial ecosystems (Marjanović et al., 2015), with elevated mycelial biomass exclusively observed in non-moisture-limited habitats (Iotti et al., 2018). This study presents the first comprehensive investigation of *T. indicum*-mediated physiological adaptations, mycorrhizosphere-associated microbial dynamics, and host root metabolic reprogramming across differential water regimes under controlled experimental conditions.

### 4.1 Mycorrhization by *Tuber indicum* affects plant physiological responses under different water regimes

Chlorophyll is an important substance involved in photosynthesis. The content of malondialdehyde can reflect the degree of plant membrane lipid peroxidation and is one of the common indicators to measure the degree of oxidative stress. Proline plays an important role in osmotic regulation in the plant cytoplasm. In this study, the results showed that the colonization of *T. indicum* significantly enhances the photosynthetic capacity of the host plant under mild and moderate drought stress, as evidenced by the increased chlorophyll concentration and reduced malondialdehyde levels. The increased concentration of Chlorophyll suggests that *T. indicum* colonization helps maintain higher photosynthetic efficiency under water-limited conditions. The reduction in malondialdehyde concentration, a marker of oxidative stress, indicates that *T. indicum* colonization mitigates oxidative damage to plant cells, thereby enhancing drought tolerance. This finding aligns with previous studies showing that plants accumulate osmoprotectants such as proline and soluble carbohydrates under drought stress to maintain protein structure and reduce damage (Saiyaremu et al., 2021; Liu et al., 2021). However, under severe drought conditions, the proline concentration in the host plant cells decreases, weakening osmotic regulation. This suggests that while *T. indicum* colonization can improve drought resistance, its effectiveness may be limited under extreme drought conditions.

### 4.2 *Tuber indicum* colonization altered mycorrhizosphere bacterial communities under different water regimes

The mycorrhizosphere bacterial communities of *T. indicum*-colonized plants showed distinct differences compared to non-mycorrhizal controls. We found that Proteobacteria, Acidobacteria, and Bacteroidetes was the three major phylum in the mycorrhizosphere of both inoculated seedlings and control group in each water regimes, which are known for their roles in nutrient cycling and plant growth promotion. (Deveau et al., 2016; Huang et al., 2023). Studies have shown that the relative abundance of Proteobacteria and Bacteroidetes of drought-sensitive varieties increased significantly under drought stress (Fan et al., 2023). Proteobacteria are highly sensitive to environmental changes due to the large number of non - dormant cells they contain, which leads to either an increase or a decrease in their abundance (Jones and Lennon, 2010). Acidobacteria is known as a plant growth promoting rhizobacteria (PGPR) that promotes the adaptation of host plant under drought stress (Naylor et al., 2017). In this study, the abundance of Acidobacteria increased in the water-stress group, demonstrating the adaptability of plant mycorrhizosphere bacteria to drought stress. These suggests that these bacteria may play a role in enhancing plant drought tolerance. The reduced bacterial diversity in the *T. indicum*-colonized group under moderate and severe drought stress may indicate a selective enrichment of beneficial bacteria that contribute to plant stress resistance. This finding is supported by previous studies showing that mycorrhizal fungi can alter the rhizosphere bacterial community structure to enhance plant resilience to environmental stress (Bulgarelli et al., 2013; Fan et al., 2023).

Bacteria have certain functions in participating in plant growth and development as well as plant metabolism (Fan et al., 2023). Meanwhile, many studies have shown that the bacterial richness and diversity of the host plant rhizosphere which colonized by *Tuber* spp. is significantly higher than those in the non-ectomycorrhizal (Wang et al., 2021; Huang et al., 2023; Berrios et al., 2024). In the present study, we also found that the rhizosphere bacterial community (Bacteroides, Patescibacteria, Firicutes and Armatimonadetes) colonized by *T. indicum* was significantly higher than non-ectomycorrhizal group. However, Li et al., 2017 reported that the bacterial diversity of *T. indicum* colonized *P. armandii* ectomycorrhizosphere soil was significantly lower compared with non-ectomycorrhizal soil. This difference may be due to the fact that the soil sample was collected at the early symbiotic stage (five month after inoculation). Furthermore, we found that the bacterial α-diversity have no difference between different water regimes in *T. indicum* colonized group. While the richness (Chao1 and Observed species) of bacterial communities in the *T. indicum* colonized group was significantly lower than that in the control group, under moderate and severe water stress. This result might be due to the fact that drought affected the root exudates of *T. indicum* mycorrhizal roots, thereby influencing the bacterial diversity (Oppenheimer-Shaanan et al., 2022).

Furthermore, the abundances of some bacteria are correlated with the physiological indicators of host plants. Numerous studies have demonstrated that certain species within the genus *Nocardioides* can promote plant growth, enhance plants’ resistance to diseases and tolerance to stress, as well as contribute to the degradation of pollutants (Cao et al., 2024; Khilyas et al., 2024; Li et al., 2024; Ma et al., 2024). In this study, *N. simplex* and *N. pyridinolyticus* had significantly positive correlation with proline, and there was a significantly positive correlation between *N. szechwanensis* and photosynthetic pigment indicators, which may imply that *N. simplex*, *N. pyridinolyticus* and *N. szechwanensis* can help plants resist drought stress by regulating the proline and photosynthetic pigments metabolism of plants.

### 4.3 *Tuber indicum* colonization effect on mycorrhizosphere metabolites under different water regimes

The formation of ectomycorrhiza has a significant impact on plant metabolites. It can regulate the production of enzymes, facilitate carbon - nitrogen exchange, and promote the synthesis of phenolic compounds and flavonoids (Saiyaremu et al., 2021; Plett et al., 2024). The most prominent observation was that uncolonized roots had many more metabolites that were enriched compared to EMF-colonized roots (Berrios et al., 2024). As we have found that there were significant differences in metabolites between plants colonized by *T. indicum* and uncolonized ones, which include the biosynthesis of secondary metabolites and amino acids, as well as in the metabolism of alanine, aspartate and glutamate, inositol phosphate, galactose, and flavonoids. These metabolic shifts suggest that *T. indicum* colonization induces changes in plant metabolism to enhance drought resistance. To better adapt to drought stress, plants have evolved a variety of complex physicochemical and metabolic mechanisms and strategies to ensure survival and reproduction (Lv et al., 2025; Singh et al., 2025). In this study, there were significant differences in metabolites between moderate and severe water stress condition and normal and mild stress conditions. Especially under the severe drought stress, the enrichment of compounds related to amino acid and sugar metabolism under severe drought stress suggests that these metabolites act as osmoprotectants, helping plants maintain cellular integrity and function under water-limited conditions. This result is consistent with the previous research findings (Abdel-Salam et al., 2018; Liu et al., 2021).

We observed that there was a significant negative correlation between trans-2-hexenal and malondialdehyde, and a significant positive correlation between trans-2-hexenal and photosynthetic pigments, whilst there is a positive correlation between *N. albus* and the secretion of trans-2-hexenal in mycorrhizosphere. To our knowledge, trans-2-hexenal has a variety of important biological functions in plants. It can not only activate the early defense signals in plant cells, including the burst of H_2_O_2_ on the plasma membrane, the directional flow of calcium ions, and the increase in cytoplasmic calcium concentration, but also serve as a volatile signaling molecule to transmit defense information between plants. This helps plants quickly respond to stresses and initiate defense mechanisms (Hao et al., 2024). This indicates that this compound may play a role in reducing oxidative stress and enhancing photosynthetic efficiency. The correlation between *Nocardioides albus* and trans-2-hexenal further supports the idea that specific bacterial taxa can influence plant metabolic responses to drought.

### 4.4 Molecular mechanisms underlying *T. indicum*-mediated drought tolerance

While our study highlights the roles of physiological adjustments, rhizosphere microbial shifts, and metabolic reprogramming in *T. indicum*-enhanced drought tolerance of *P. armandii*, we acknowledge that the molecular mechanisms driving these changes remain to be fully elucidated. Metabolomic analyses identified key differential metabolites (e.g., trans-2-hexenal, carbohydrates, and amino acids) and their associations with drought-responsive traits, but these metabolic phenotypes are downstream consequences of upstream molecular events, including gene transcription and protein expression.

Emerging transcriptomic and proteomic studies on mycorrhizal symbiosis under drought provide valuable insights into potential regulatory pathways. For instance, transcriptome analyses of ectomycorrhizal plants have revealed upregulation of genes involved in abscisic acid (ABA) biosynthesis, antioxidant enzyme production (e.g., superoxide dismutase, peroxidase), and osmolyte synthesis (e.g., proline, trehalose) under water stress (Abdalla et al., 2023; Bahadur et al., 2019; Zhang et al., 2025). These genes likely contribute to reduced membrane lipid peroxidation (lower malondialdehyde) and enhanced osmotic adjustment, consistent with our physiological data. Additionally, proteomic studies have linked mycorrhization to increased expression of photosynthesis-related proteins (e.g., chlorophyll-binding proteins) and transporters for water and nutrients, which may explain the higher photosynthetic pigment contents observed in T. indicum-colonized seedlings (Shi et al., 2022).

With respect to specific metabolites, trans-2-hexenal—identified here as a key mediator of drought tolerance—has been associated with transcriptional activation of defense-related genes (e.g., WRKY and MYC2) via signaling pathways involving calcium ions and reactive oxygen species (Zhang et al., 2025). The positive correlation between N. albus and trans-2-hexenal further suggests that microbial signals may modulate host gene expression, a mechanism supported by studies showing rhizobacteria-induced transcriptional changes in plant stress-responsive genes (Bharti et al., 2016).

### 4.5 Limitations

This study provides insights into the role of *T. indicum* colonization in enhancing drought tolerance of *P. armandii* through physiological, microbial, and metabolic adjustments, but several limitations should be acknowledged.

The experiment was conducted under strictly controlled greenhouse conditions with artificial water regime manipulation. While this allowed for precise control of variables (e.g., temperature, humidity, and soil substrate), the environmental complexity of natural ecosystems-such as fluctuating climatic factors, soil heterogeneity, and interactions with diverse microbial communities-was not fully captured. Thus, field validation in drought-prone areas is essential to verify whether the observed effects of *T. indicum* colonization can be generalized to real-world ecological contexts, particularly under long-term and variable drought stress.

The metabolomic and microbial community analyses were based on samples collected at a single time point (40 days after drought treatment). This static snapshot limits our ability to capture dynamic regulatory processes, such as temporal changes in metabolite biosynthesis, bacterial community succession, or the timing of key physiological adjustments during the progression of drought stress. Time-series sampling would provide a more comprehensive understanding of how *T. indicum* modulates host responses and rhizosphere interactions over the course of drought exposure.

While correlation analyses identified potential roles of key bacteria (e.g., Nocardioides albus) and metabolites (e.g., trans-2-hexenal) in enhancing drought tolerance, their functional significance remains speculative. For example, the positive association between *N. albus* and trans-2-hexenal, and the links between trans-2-hexenal and photosynthetic pigment/malondialdehyde levels, require direct validation through experimental manipulation. Functional assays-such as bacterial inoculation experiments, metabolite exogenous application, or knockout/overexpression of genes involved in trans-2-hexenal biosynthesis-are necessary to confirm causal relationships and elucidate the mechanistic basis of these correlations.

These limitations highlight the need for future studies to integrate field validation, time-series analyses, and functional experiments to strengthen the robustness and generalizability of our findings.

## 5 Conclusions

It is the first time to demonstrate the impact of *T. indicum* colonization on the drought resistance of host plant under controlled condition. This is the first study to systematically reveal the synergistic regulation of *T. indicum* on host physiology, rhizosphere metabolites, and bacterial communities under drought stress. The colonization of *T. indicum* promoted the accumulation of proline content under drought stress, reduced the malondialdehyde content under severe drought stress, and helped the plants resist the damage caused by drought stress. It achieved this by stabilizing the structure of the rhizosphere microbial community and promoting amino acid and sugar metabolism to obtain a large number of osmotic adjustment substances. *N. albus* has a significant positive correlation with trans-2-hexenal, and trans-2-hexenal shows a significant negative correlation with the malondialdehyde content and a significant positive correlation with the content of photosynthetic pigments in the host. These correlations imply that *N. albus* may play an important role in plant drought resistance. These results can provide references for the application of truffles in plant drought resistance.

## Data Availability

The 16S rRNA raw sequence data used in this study have been deposited in the NCBI Sequence Read Archive database under the bioproject identifier PRJNA1309842, which are publicly accessible online.

## Supplementary material

Supplementary Figure 1 The relative abundance of TOP 10 bacterial groups at the phylum level in the rhizosphere of mycorrhizal and non-mycorrhizal (CK) *Pinus armandii* seedlings under different water regimes. well, 75-80% water holding capacity (WHC); mild, mild drought stress, 60-65% WHC; moderate, moderate drought stress, 40-50% WHC; severe, severe drought stress, 25-35% WHC.

Supplementary Figure 2 Impact of *Tuber indicum* colonization and water regimes on rhizosphere bacterial compositions of *Pinus armandii* seedlings at the genus level. well, 75-80% water holding capacity (WHC); mild, mild drought stress, 60-65% WHC; moderate, moderate drought stress, 40-50% WHC; severe, severe drought stress, 25-35% WHC.

Supplementary Figure 3 Unique and shared bacterial operation taxonomic units (OTUs) influenced by *Tuber indicum* colonization of *Pinus armandii* seedlings under different water regimes. well, 75-80% water holding capacity (WHC); mild, mild drought stress, 60-65% WHC; moderate, moderate drought stress, 40-50% WHC; severe, severe drought stress, 25-35% WHC. CK, the control (non-inoculated) samples.

Supplementary Figure 4 Heatmap showing the Top 20 bacterial OTUs enriched in the rhizosphere of mycorrhizal and non-mycorrhizal *Pinus armandii* seedlings under different water regimes. well, 75-80% water holding capacity (WHC); mild, mild drought stress, 60-65% WHC; moderate, moderate drought stress, 40-50% WHC; severe, severe drought stress, 25-35% WHC. CK, the control (non-inoculated) samples.

Supplementary Figure 5 Distribution of different types of all annotated root metabolites.

Supplementary Figure 6 Distribution of different types of all annotated root metabolites.

Supplementary Figure 7 Partial least squares-discriminant analysis (PLS-DA) of metabolites in the *T. indicum* colonization group and control group of *Pinus armandii* seedlings. “Ti vs CK”**(A)**, and “four water regimes group”**(B)**.

Supplementary Figure 8
**(A-D)** Differentially expressed root metabolites affected by *Tuber indicum* colonization of *Pinus armandii* seedlings under different water regimes. well, 75-80% water holding capacity (WHC); mild, mild drought stress, 60-65% WHC; moderate, moderate drought stress, 40-50% WHC; severe, severe drought stress, 25-35% WHC. CK, the control (non-inoculated) samples.

Supplementary Figure 9 Cluster heatmap showing differentially expressed root metabolites impacted by *Tuber indicum* colonization of *Pinus armandii* seedlings under different water regimes. well, 75-80% water holding capacity (WHC); mild, mild drought stress, 60-65% WHC; moderate, moderate drought stress, 40-50% WHC; severe, severe drought stress, 25-35% WHC. CK, the control (non-inoculated) samples.
