# Supplementary figures and images for: Tuber indicum colonization enhances plant drought tolerance by modifying physiological, rhizosphere metabolic and bacterial community responses in Pinus armandii
Source: Front Plant Sci. 2025 Oct 28;16:1642071. doi: 10.3389/fpls.2025.1642071 (PMC12604364; doi:10.3389/fpls.2025.1642071)

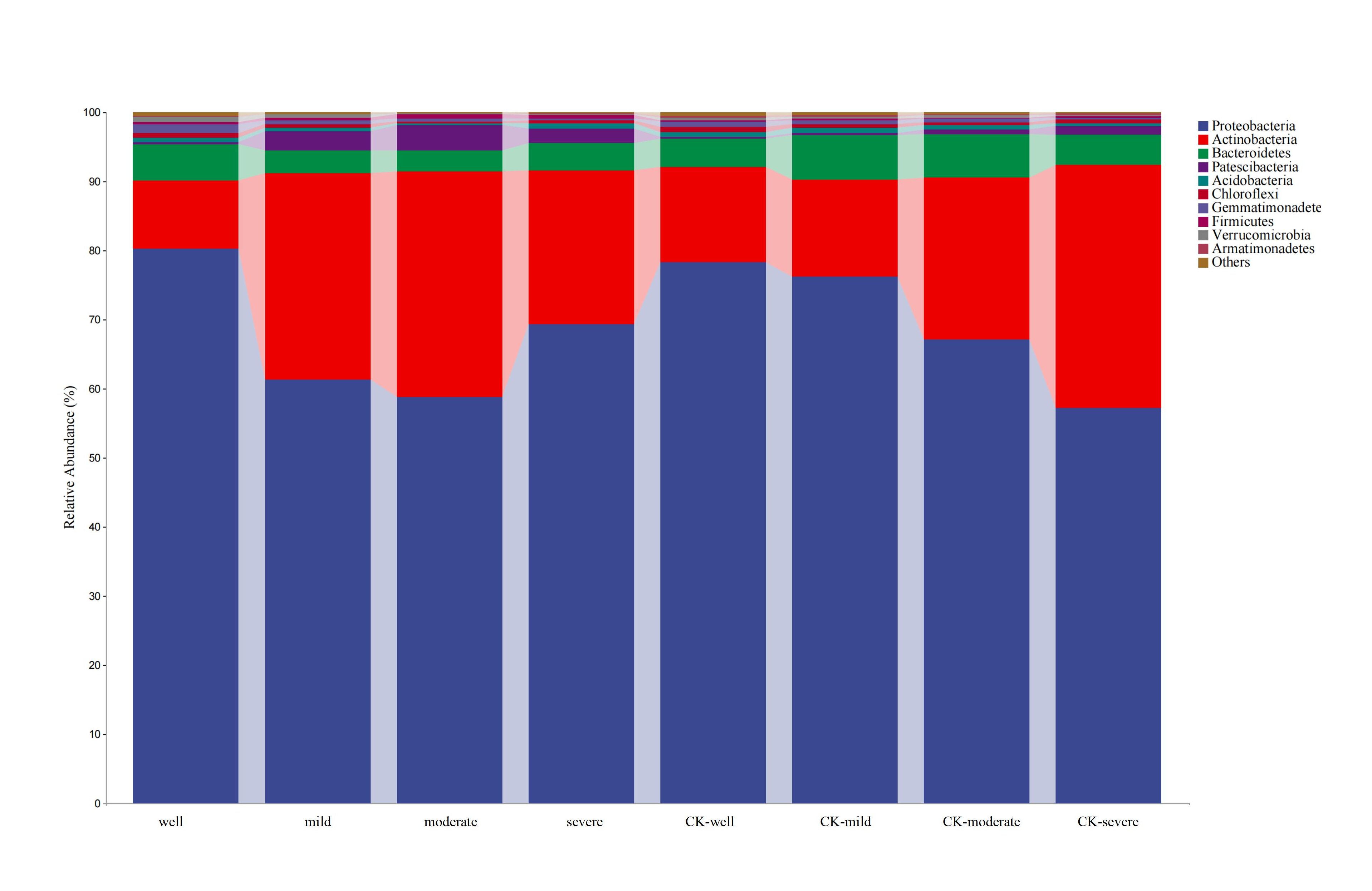

Supplement: Supplementary Figure 1 — The relative abundance of TOP 10 bacterial groups at the phylum level in the rhizosphere of mycorrhizal and non-mycorrhizal (CK) Pinus armandii seedlings under different water regimes. well, 75-80% water holding capacity (WHC); mild, mild drought stress, 60-65% WHC; moderate, moderate drought stress, 40-50% WHC; severe, severe drought stress, 25-35% WHC. [file Image1.jpeg]

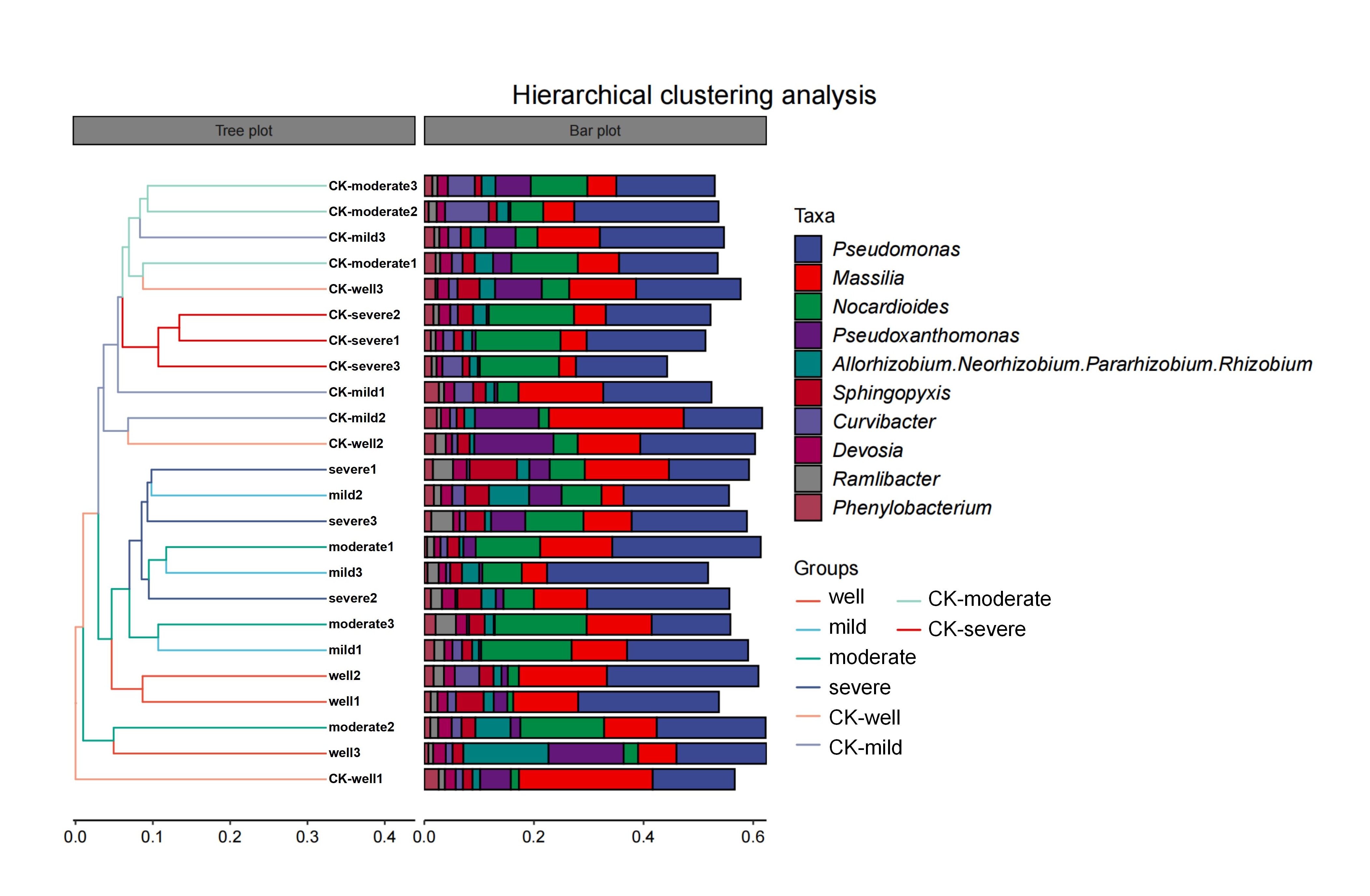

Supplement: Supplementary Figure 2 — Impact of Tuber indicum colonization and water regimes on rhizosphere bacterial compositions of Pinus armandii seedlings at the genus level. well, 75-80% water holding capacity (WHC); mild, mild drought stress, 60-65% WHC; moderate, moderate drought stress, 40-50% WHC; severe, severe drought stress, 25-35% WHC. [file Image2.jpeg]

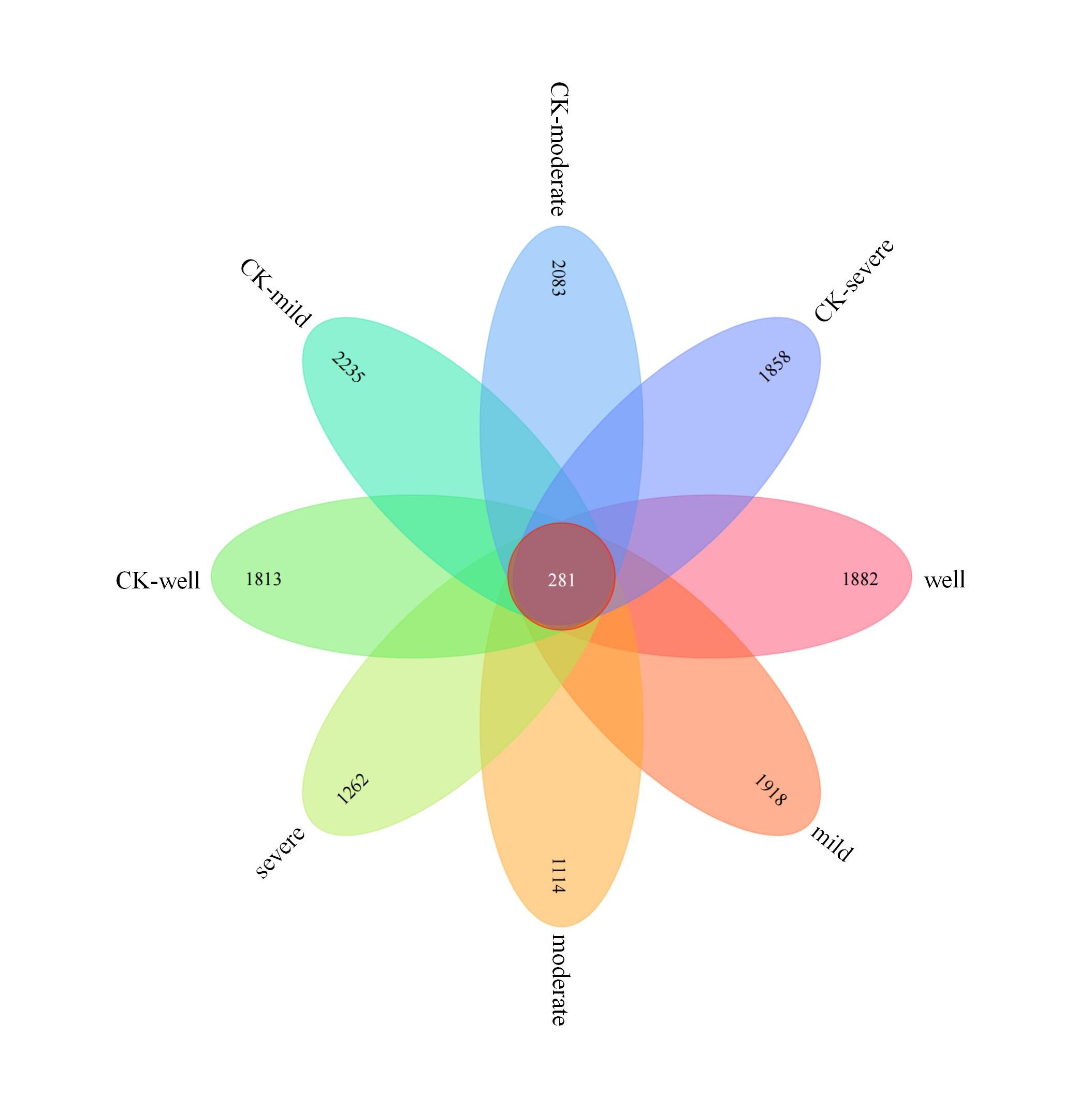

Supplement: Supplementary Figure 3 — Unique and shared bacterial operation taxonomic units (OTUs) influenced by Tuber indicum colonization of Pinus armandii seedlings under different water regimes. well, 75-80% water holding capacity (WHC); mild, mild drought stress, 60-65% WHC; moderate, moderate drought stress, 40-50% WHC; severe, severe drought stress, 25-35% WHC. CK, the control (non-inoculated) samples. [file Image3.jpeg]

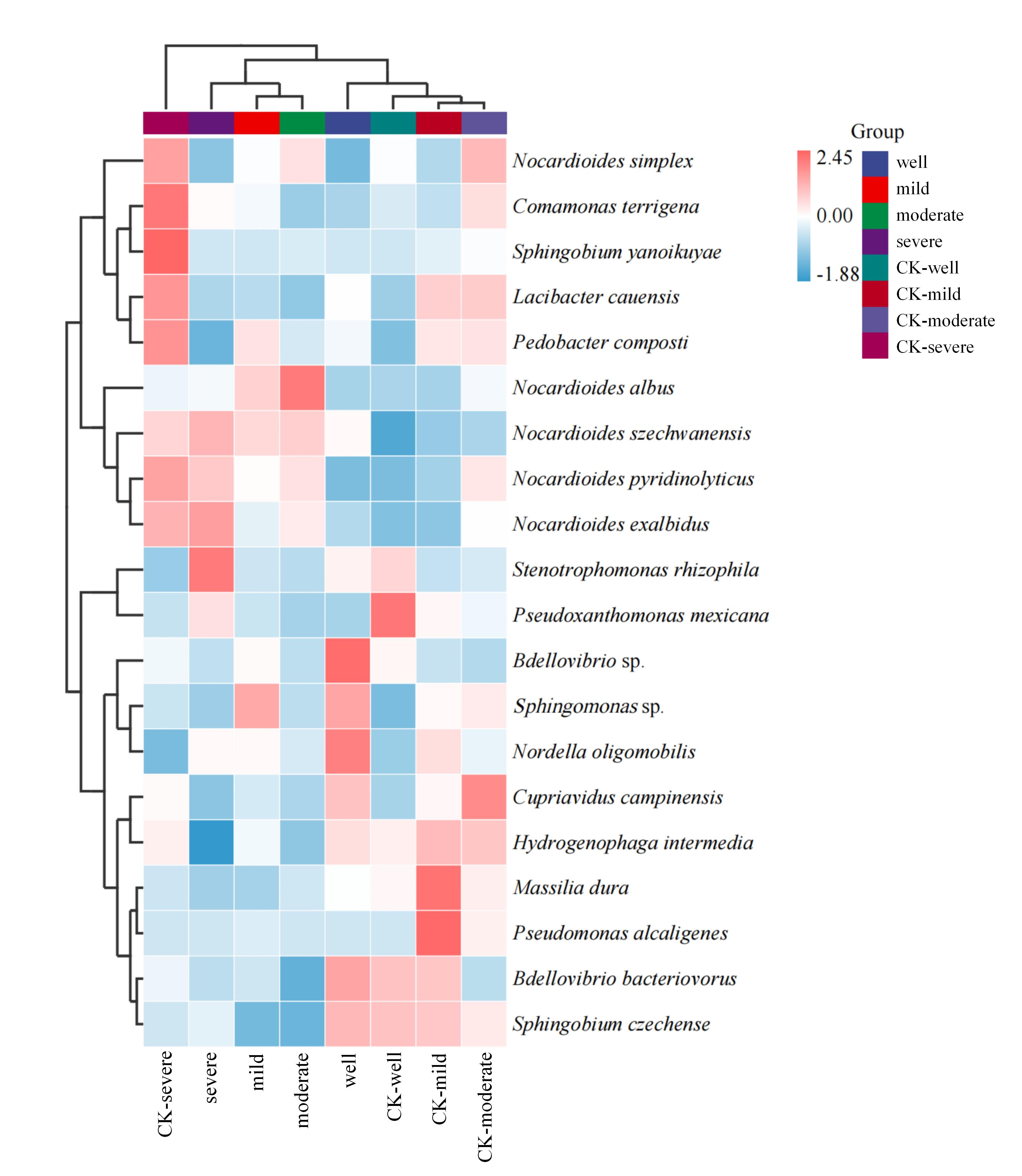

Supplement: Supplementary Figure 4 — Heatmap showing the Top 20 bacterial OTUs enriched in the rhizosphere of mycorrhizal and non-mycorrhizal Pinus armandii seedlings under different water regimes. well, 75-80% water holding capacity (WHC); mild, mild drought stress, 60-65% WHC; moderate, moderate drought stress, 40-50% WHC; severe, severe drought stress, 25-35% WHC. CK, the control (non-inoculated) samples. [file Image4.jpeg]

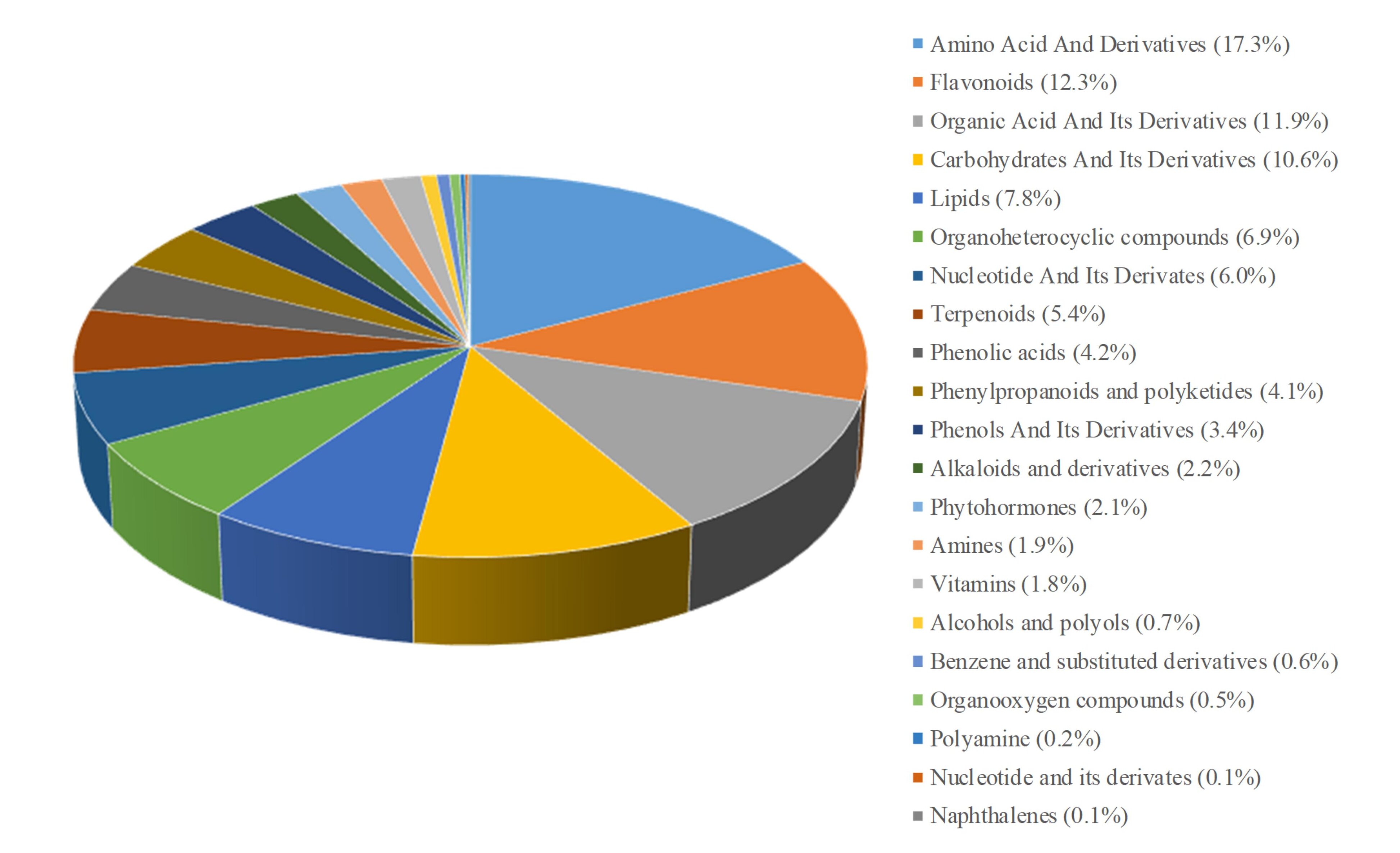

Supplement: Supplementary Figure 5 — Distribution of different types of all annotated root metabolites. [file Image5.jpeg]

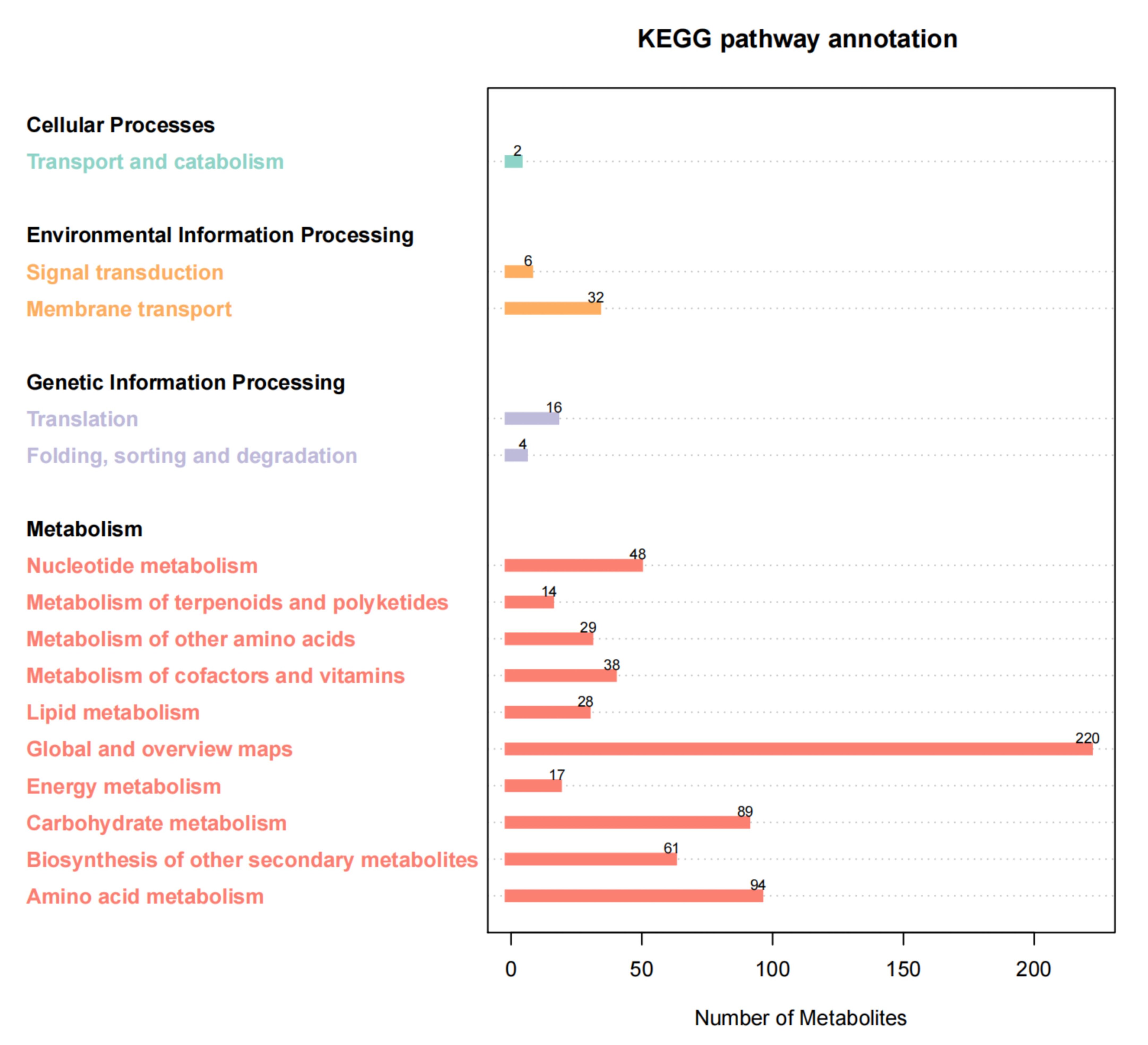

Supplement: Supplementary Figure 6 — Distribution of different types of all annotated root metabolites. [file Image6.jpeg]

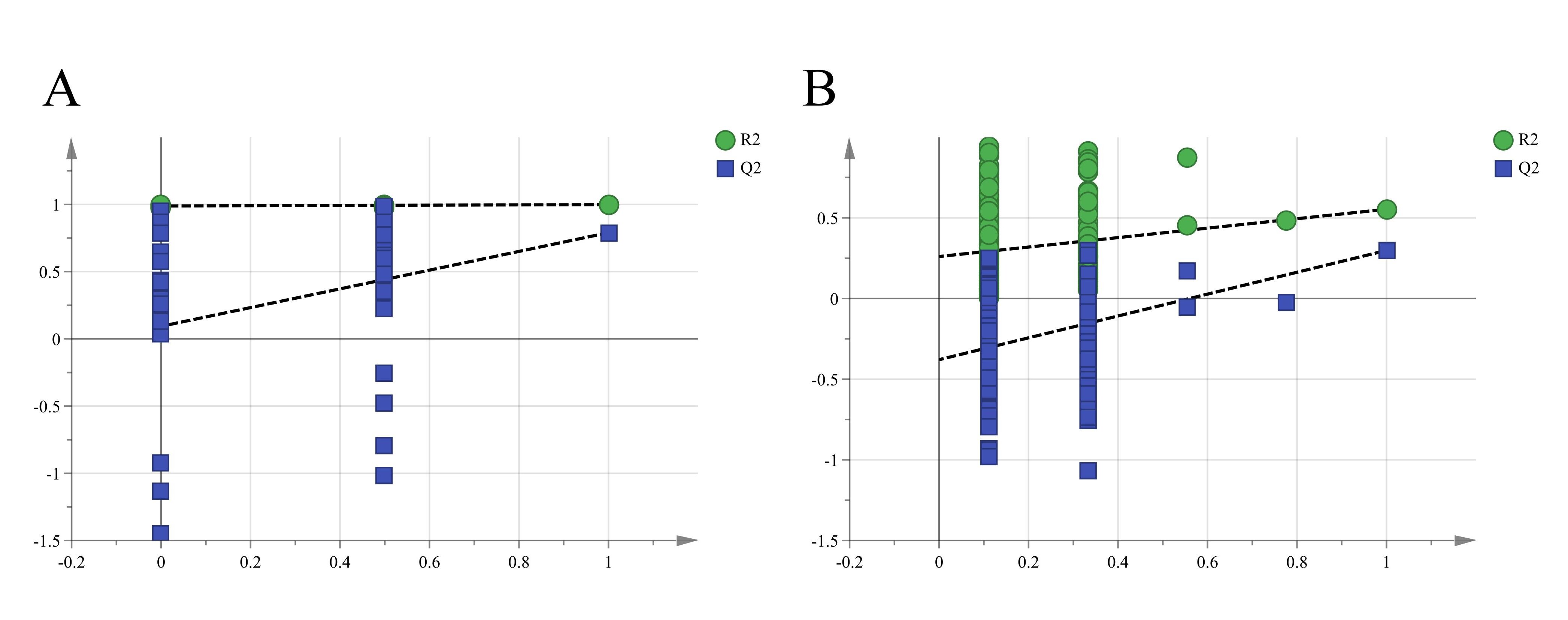

Supplement: Supplementary Figure 7 — Partial least squares-discriminant analysis (PLS-DA) of metabolites in the T. indicum colonization group and control group of Pinus armandii seedlings. “Ti vs CK”(A), and “four water regimes group”(B). [file Image7.jpeg]

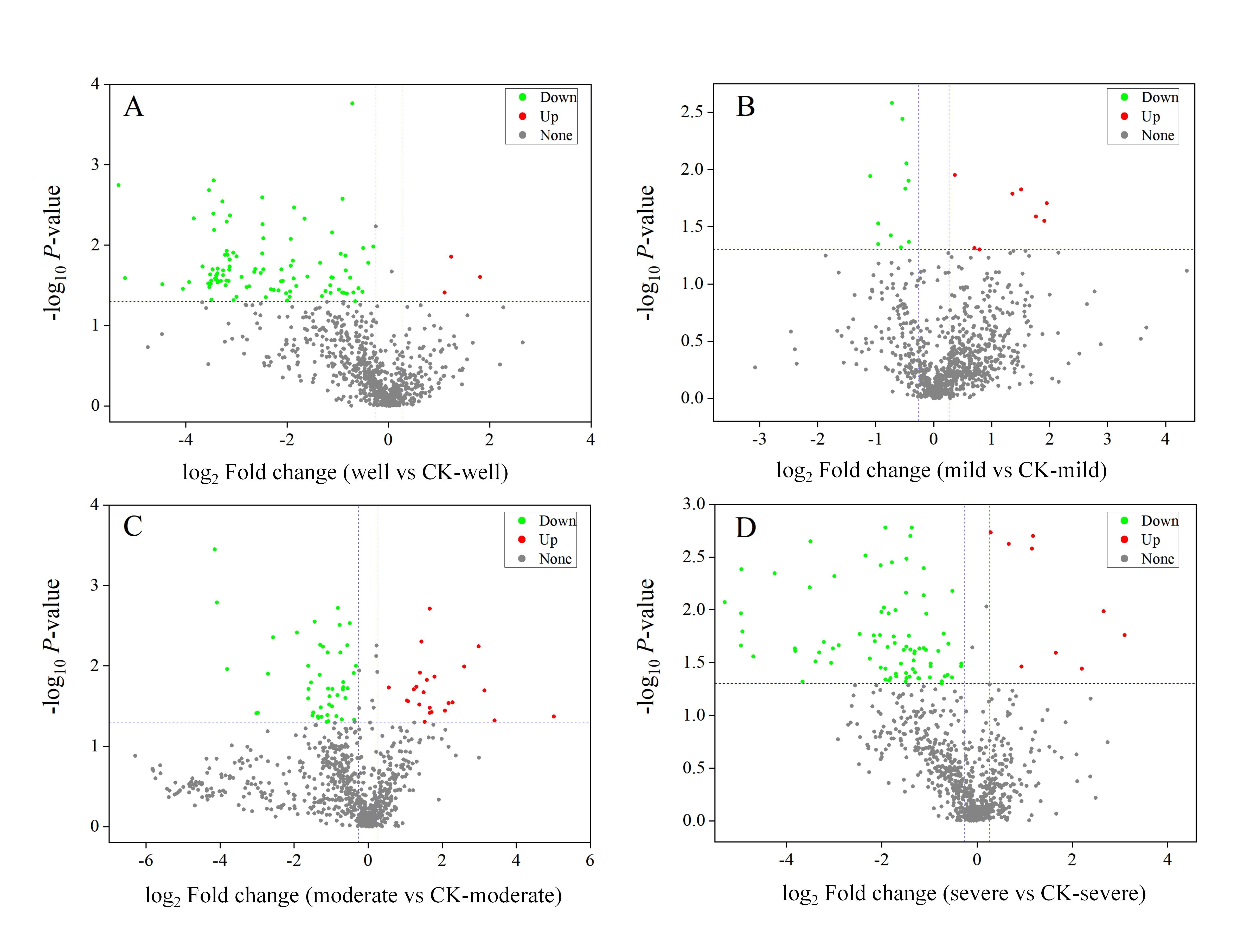

Supplement: Supplementary Figure 8 — (A-D) Differentially expressed root metabolites affected by Tuber indicum colonization of Pinus armandii seedlings under different water regimes. well, 75-80% water holding capacity (WHC); mild, mild drought stress, 60-65% WHC; moderate, moderate drought stress, 40-50% WHC; severe, severe drought stress, 25-35% WHC. CK, the control (non-inoculated) samples. [file Image8.jpeg]

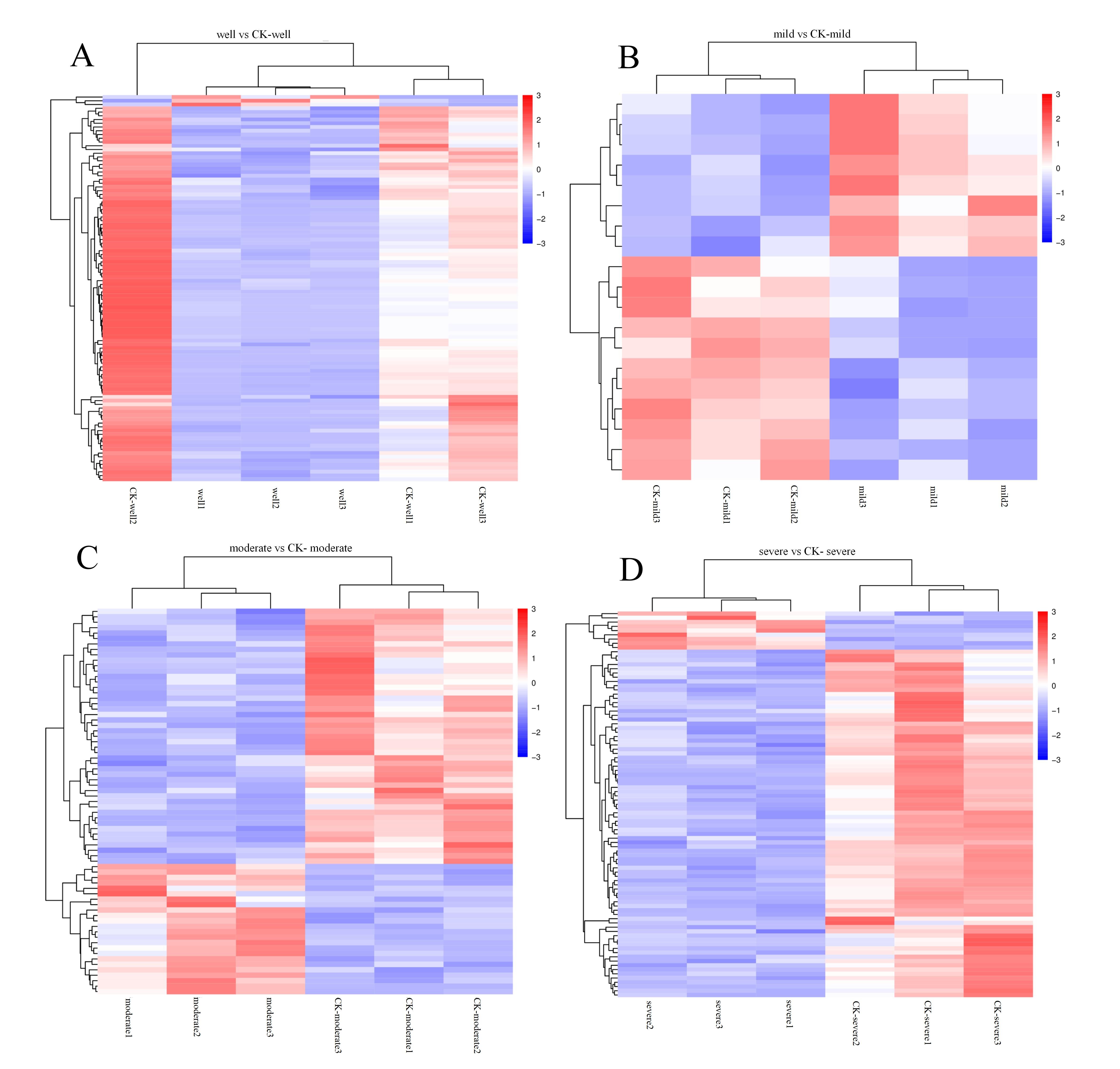

Supplement: Supplementary Figure 9 — Cluster heatmap showing differentially expressed root metabolites impacted by Tuber indicum colonization of Pinus armandii seedlings under different water regimes. well, 75-80% water holding capacity (WHC); mild, mild drought stress, 60-65% WHC; moderate, moderate drought stress, 40-50% WHC; severe, severe drought stress, 25-35% WHC. CK, the control (non-inoculated) samples. [file Image9.jpeg]
